# Specific Phosphorylation of Histone Demethylase KDM3A Determines Target Gene Expression in Response to Heat Shock

**DOI:** 10.1371/journal.pbio.1002026

**Published:** 2014-12-23

**Authors:** Mo-bin Cheng, Yan Zhang, Chun-yu Cao, Wei-long Zhang, Ye Zhang, Yu-fei Shen

**Affiliations:** 1 State Key Laboratory of Medical Molecular Biology, Department of Biochemistry and Molecular Biology, Institute of Basic Medical Sciences, Chinese Academy of Medical Sciences and School of Basic Medicine, Peking Union Medical College, Beijing, China; 2 Cancer Institute and Hospital, Chinese Academy of Medical Sciences and Peking Union Medical College, Beijing, China; U.T.M.D. Anderson Cancer Center, United States of America

## Abstract

Histone lysine (K) residues, which are modified by methyl- and acetyl-transferases, diversely regulate RNA synthesis. Unlike the ubiquitously activating effect of histone K acetylation, the effects of histone K methylation vary with the number of methyl groups added and with the position of these groups in the histone tails. Histone K demethylases (KDMs) counteract the activity of methyl-transferases and remove methyl group(s) from specific K residues in histones. KDM3A (also known as JHDM2A or JMJD1A) is an H3K9me2/1 demethylase. KDM3A performs diverse functions via the regulation of its associated genes, which are involved in spermatogenesis, metabolism, and cell differentiation. However, the mechanism by which the activity of KDM3A is regulated is largely unknown. Here, we demonstrated that mitogen- and stress-activated protein kinase 1 (MSK1) specifically phosphorylates KDM3A at Ser264 (p-KDM3A), which is enriched in the regulatory regions of gene loci in the human genome. p-KDM3A directly interacts with and is recruited by the transcription factor Stat1 to activate p-KDM3A target genes under heat shock conditions. The demethylation of H3K9me2 at the Stat1 binding site specifically depends on the co-expression of p-KDM3A in the heat-shocked cells. In contrast to heat shock, IFN-γ treatment does not phosphorylate KDM3A via MSK1, thereby abrogating its downstream effects. To our knowledge, this is the first evidence that a KDM can be modified via phosphorylation to determine its specific binding to target genes in response to thermal stress.

## Abstract 2

Phosphorylation of histone demethylase KDM3A in response to thermal stress enables its specific recruitment to target genes by Stat1.

## Author Summary

Histone methylation regulates gene expression and can have drastic consequences for health if the process is defective. Histone lysine demethylases (KDMs) counteract the activity of methyl-transferases and remove methyl group(s) from histones. KDM3A is a H3K9me2/1 demethylase that performs diverse functions via the regulation of its target genes, which are involved in spermatogenesis, metabolism, and cell differentiation. However, the mechanisms underlying KDM3A regulation of specific genes at specific times are largely unknown. Here we found that a physiological stress—elevated temperature—induces KDM3A phosphorylation in human cells via the MSK1 kinase. This phosphorylated form of KDM3A directly interacts with the transcription factor Stat1, which enables Stat1 to recruit KDM3A to Stat1-binding sequences at the promoters of specific target genes. KDM3A then acts to demethylate H3K9me2/1 at these targets, thereby causing specific gene expression in response to the thermal stress. We conclude that heat shock can affect the expression of many genes in human cells via a novel activation mechanism that is centered around the phosphorylation of KDM3A.

## Introduction

Histone modifications, such as methylation and acetylation, regulate RNA synthesis [1],[2]. Unlike the activating impact of acetylation, the methylation of lysine residues in histones can exert either an activating or a repressive effect on genes, depending on the number of methyl groups that are added and the position of the lysine residue in the histone tail [3]. For example, the di- or tri-methylation of lysine (K) 9 on histone (H) 3 (H3K9me2/3), H3K27me2/3, and H4K20me3 is repressive, whereas that of H3K4me3 and H3K36me3 enhances the transcription of their target genes [4]–[6]. A major breakthrough in this field was the discovery that the methylation of histone tails is a reversible process. This discovery was based on the identification of two classes of histone lysine demethylases (KDMs), namely the FAD-dependent amine oxidase LSD1 [7] and the Jumonji C (JmjC) domain demethylases, a family of Fe^2+^- and 2-oxoglutarate-dependent KDMs [8].

Among the JmjC domain demethylases, KDM3A (also known as JHDM2A or JMJD1A) was first identified as a testis-enriched zinc finger protein that is highly expressed in male germ cells and is involved in germ cell development [9]. KDM3A was later identified as an H3K9me2/1 demethylase that activates the expression of the androgen receptor (AR) gene via an androgen-dependent pathway [10]. Furthermore, KDM3A has been demonstrated to regulate genes that are involved in spermatogenesis [11],[12], metabolism [13], and cell differentiation [14]. With such a broad functional diversity, the mechanism by which KDM3A regulates the appropriate gene(s) in vivo at the appropriate time and targets the appropriate element is of great interest.

Post-translational protein modification is very important for determining the function of proteins, including JmjC domain-containing proteins such as PHF8, which is phosphorylated by cyclin-dependent kinases (CDK), inducing the dissociation of PHF8 from chromatin [15]. PHF2 is enzymatically inactive in isolation, but PKA-phosphorylated PHF2 in complex with ARID5B displays H3K9Me2 demethylase activity [16]. PKCα–phosphorylated LSD1 forms a complex with CLOCK:BMAL1 to facilitate E-box-mediated transcriptional activation [17]. However, it is unknown whether KDM3A is phosphorylated, and the consequences of such a modification are also unknown.

In this study, we demonstrate that MSK1 is activated and specifically phosphorylates KDM3A at Ser264 under heat shock. The phosphorylated KDM3A (p-KDM3A) is enriched at the regulatory regions of gene loci and co-localizes with Stat1 in the human genome. Extensive experiments indicate that p-KDM3A directly interacts with and is recruited by Stat1 to mediate chromatin remodeling and the expression of its target genes in response to heat shock.

## Results

### KDM3A Is Phosphorylated at Ser264 by MSK1

**Figure 1:**
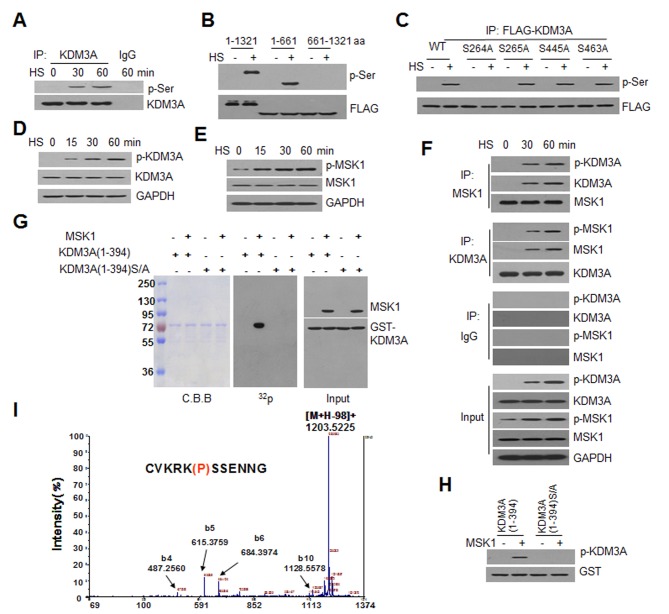
KDM3A is phosphorylated at S264 by MSK1 under HS conditions. KDM3A phosphorylation was determined via co-IP and western blot assays of Jurkat cells that were treated with heat shock at 42°C (HS) for 0–60 min. (**A**) IP was performed on whole cell extracts (WCE) using an antibody against KDM3A or IgG (as a negative control). The antibodies that were used for western blot, including p-Ser and KDM3A, are shown on the right. (**B**) The truncated FLAG-KDM3A constructs were transfected into Jurkat cells, which were then treated with (+) or without HS (-). The WCE were immunoprecipitated using the FLAG antibody. The FLAG-tagged fragments of KDM3A were as follows: 1-1321 aa, 1-661 aa, and 661-1321 aa. The antibodies used for western blot are shown on the right. (**C**) IP assay of wild-type and at S264A, S265A, S445A, and S463A mutant FLAG-tagged KDM3A-transfected cells treated with (+) or without HS (-). (**D**) Western blot using an antibody against p-KDM3A-S264 at the indicated time. The antibodies against KDM3A and GAPDH were used as positive and loading controls, respectively. (**E**) Western blot of p-MSK1 in Jurkat cells that were subjected to HS for 0, 15, 30, or 60 min. The p-MSK1 level was determined using an antibody that was specific for MSK1 phosphorylated at S376. The MSK1 and GAPDH antibodies were used as controls. (**F**) p-KDM3A interacts with p-MSK1 in heat-shocked cells. Co-IP assays were performed using an anti-MSK1 antibody followed by western blot using antibodies for p-KDM3A, KDM3A, and MSK1, and those proteins that immunoprecipitated with anti-KDM3A were subjected to western blot for p-MSK1, MSK1, and KDM3A. (**G and H**) In vitro kinase assays. Recombinant MSK1 was incubated in purified GST-KDM3A (1-394 aa) or the corresponding S264A mutant. Then, the reaction mixtures were separated via SDS-PAGE. The ^32^P-labeled proteins were visualized via autoradiography (central panel). Western blots were performed using antibodies against MSK1 and GST (right panel), and the level of KDM3A-GST was assessed via Coomassie staining (left panel) (G). A western blot was performed on MSK1 added to (+) WCE from cells that were transfected with wild-type or S/A mutant KDM3A(1-394). The specific antibody against p-KDM3A was used for western blot, and GST was used as the input (H). (**I**) Mass spectrometric analysis of the synthesized peptide KDM3A(260-269) (insert panel) phosphorylated using recombinant MSK1. The difference between the b5 ion of K and the b6 ion of serine (S) in the spectrum indicates that S264 was phosphorylated in the peptide. b ion: fragmentation ion containing the N-terminus of the peptide.

Histone modifications are recognized by specific proteins, including transcription factors (TFs), thereby mediating functional signaling to affect chromatin condensation or remodeling near target genes [2],[18],[19]. Methylated H3K9, a repressive histone mark, must be recognized and demethylated during the initiation of gene activation. Among the identified KDMs, KDM3A was the only KDM that targeted an IFNγ-activated sequence (GAS) in heat-shocked Jurkat cells (S1 Figure). Using an antibody against pan-phosphorylated serine (p-Ser) to detect the proteins immunoprecipitated for phosphorylated KDM3A, we found that KDM3A was phosphorylated after 30 or 60 min of heat shock at 42°C (the treatment of cells at 42°C for 60 min is generally defined as “heat shock” or abbreviated as “HS” in this study; it should be otherwise indicated when a shorter incubation time is applied) (Fig. 1A). This phosphorylation occurred within the first 661 aa of the N-terminus of KDM3A (Fig. 1B). Analysis of mutants in which serine was substituted with alanine at 264, 265, 445, and 463 aa of KDM3A revealed that only the S264A mutant abrogated the HS-induced phosphorylation of KDM3A (Fig. 1C). Next, we generated an antibody against a serine-phosphorylated peptide (cVKRK(p)SSENNG) and verified its efficacy via western blot (S2 Figure). Phosphorylated Ser264-KDM3A (p-KDM3A) was confirmed to be specifically induced under HS (Fig. 1D).

To explore the upstream kinase responsible for KDM3A phosphorylation under heat shock, mitogen- and stress-activated protein kinase 1 (MSK1) was considered as the most likely candidate because Jil1, the *Drosophila* ortholog of human MSK1, is activated in response to heat shock [20]. Because the activation of MSK1 can be identified based on its phosphorylation at S376 (p-MSK) [21], an antibody against p-MSK was used. An increased level of p-MSK was detected following extended incubation of the cells under HS (Fig. 1E). In co-IP assays with antibody targeting either MSK1 or KDM3A, co-IP of KDM3A and MSK1 in their phosphorylated forms was found only under HS. In contrast, the non-phosphorylated forms of MSK1 and KDM3A were unable to interact with one another under physiological condition (Fig. 1F). Furthermore, this interaction in heat-shocked cells was not affected by introducing either a dominant negative mutant of MSK1 or the S264A mutant of KDM3A (S3 Figure).

Next, we analyzed the specificity of activated MSK1 for KDM3A via an in vitro kinase assay using γ-^32^P-ATP to label the phosphorylated substrate. We demonstrated that only the GST-fused wild-type N-terminal KDM3A (1-394 aa), but not the S264A mutant (S/A), was phosphorylated by MSK1 based on ^32^P labeling (central panel of Fig. 1G). Then, MSK1 was incubated in the two GST-fused KDM3A protein fragments as described above, resulting in the specific phosphorylation of wild-type but not mutant KDM3A in vitro (Fig. 1H). Furthermore, we performed an in vitro kinase assay followed by mass spectrometric analysis to determine the specific target serine of MSK1 between the two successive serine residues at 264 and 265 aa in the synthesized KDM3A peptide (Fig. 1I). These in vitro data demonstrated that MSK1 specifically phosphorylates S264 of KDM3A.

### p-KDM3A Preferentially Targets Consensus Stat1-Binding Regions in the Human Genome

**Figure 2:**
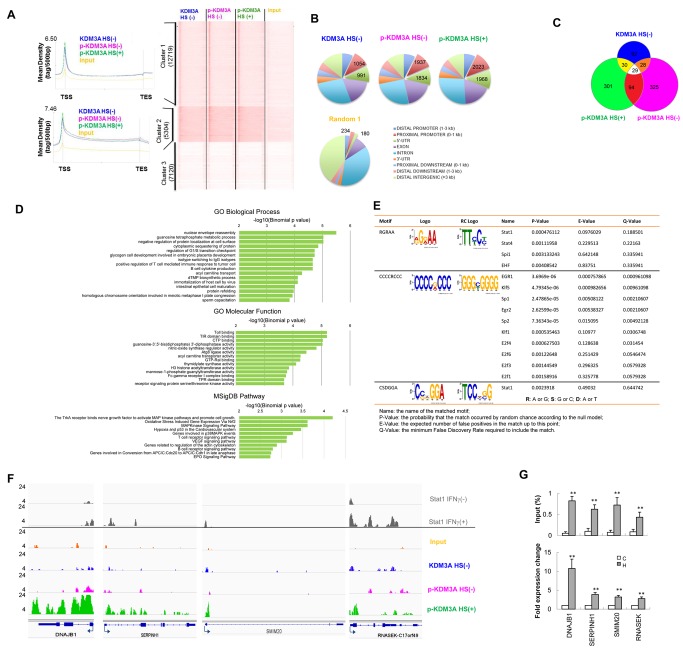
The targets of p-KDM3A in the human genome. (**A**) *Right*, Meta Gene profiles of KDM3A binding to gene loci from the TSS to the TTS. *Left*, The color intensity represents the tag count, which is standardized across the gene groups for each ChIP-seq dataset. (**B**) Pie chart of KDM3A HS(-), p-KDM3A HS(-), p-KDM3A HS(+), and random occupancies across the genome. (**C**) The Venn diagram shows the binding regions of KDM3A, p-KDM3A HS(-), and p-KDM3A HS(+) for the Jurkat cells. (**D**) GO analysis of HS-induced p-KDM3A targets using GREAT. The control analyses of KDM3A and p-KDM3A without HS treatment are shown in S5 Figure. (**E**) Motif analysis of the p-KDM3A-enriched regions using MEME. The three most distinct identified motifs are shown. (**F**) Representative ChIP-seq tracks for KDM3A and p-KDM3A on *DNAJB1, SERPIH1, SMIM20*, and *RNASEK* in Jurkat cells with or without HS treatment. The *x*-axis indicates the genomic location, and the *y*-axis represents the normalized ChIP-seq signal density. The binding peaks of Stat1 induced in HeLa S3 cells that were treated with (+) or without (-) IFN-γ were taken from Robert et al. [27] and are shown on the top two rows. (**G**) ChIP-qPCR for changes in the percentage relative to the input (top) and the fold-change in mRNA expression of the indicated genes that were induced by HS treatment. Data are mean ± SD (**p<0.01).

To determine the effect of S264 phosphorylation on KDM3A, the demethylase activity of this enzyme was examined in vitro. However, no clear changes in the activity of KDM3A with or without S264 phosphorylation were detected (S4 Figure). Then, chromatin immunoprecipitation sequence (ChIP-seq) was performed to determine the global occupancy of p-KDM3A. Chromatin fragments were immunoprecipitated using an antibody against p-KDM3A from Jurkat cells subjected to HS (+) or not (-) or using a native KDM3A antibody from Jurkat cells not subjected to HS. A heat map containing more than 25,000 elements (gene promoters) was generated using seqMINER [22], and the results presented in four rows based on the antibody used and the heat-shock status. These elements were separated into three clusters, consisting of 12,719 elements in cluster 1 (top), 5,304 elements in cluster 2 (middle), and 7,120 elements in cluster 3 (bottom) (right panel, Fig. 2A). The MetaGene profiles indicated that the reads were enriched at the transcription start site (TSS) in cluster 1 genes, whereas both the TSS and the body of the genes were enriched in those of cluster 2 (top and middle, left panel, Fig. 2A). We analyzed all of the significant peaks in each sequencing sample using SICER V1.1 [23]. The percentages of the peaks of p-KDM3A that occupied the 2,700-MB mappable genome were 0.49% (HS-) and 0.42% (HS+), and their distributions across the genome are shown in a pie chart (Fig. 2B and S1 Table). The peaks were significantly enriched in the upstream regulatory region (approximate 10-fold, all *p*<1×10^−100^). By screening the differential SICER intervals near gene promoters (from −5 kb to approximately +2 kb) (FDR threshold 10^−20^), KDM3A and the non-treated or heat-shocked p-KDM3A target genes were identified, as shown in the Venn diagrams (Fig. 2C and listed in S2 Table). Gene Ontology (GO) and MSigDB Pathway analyses were performed on the target genes using GREAT 2.0.2 [24] (Fig. 2D and S5 Figure).

Next, we performed a TF motif analysis of the p-KDM3A-binding regions under HS using MEME [25],[26] and found that two of the three most common motifs (RGRAA and CSDGGA) correspond to Stat1-binding sites, indicating the genomic co-localization of p-KDM3A with Stat1 (Fig. 2E, S6 Figure, and S3 Table). Then, we determined the nearest gene locus in the top 68 sites of p-KDM3A binding that displayed the most significant difference between the HS and control conditions (S4 Table) to determine the binding peaks of p-KDM3A at four gene loci, *DNAJB1, SERPINH1, SMIM20*, and *RNASEK*, each of which is on a distinct chromosome in Jurkat cells (Fig. 2F, bottom panel). In addition, profiles of the Stat1-binding peaks in HeLa S3 cells treated with or without IFN-γ [27] were used as a reference (top panel).

To further illustrate the relationships between p-KDM3A occupancy and the expression of selected genes, ChIP-quantitative PCR (ChIP-qPCR) and reverse transcription quantitative PCR (RT-qPCR) were performed. The data demonstrated that the occupancy of p-KDM3A at all four gene loci examined (top panel, Fig. 2G) and the mRNA expression of all of these genes were enhanced under HS (bottom panel, Fig. 2G), suggesting a correlation between these two events in heat-shocked cells.

### p-KDM3A Interacts with Stat1 in Heat-Shocked Jurkat Cells

**Figure 3:**
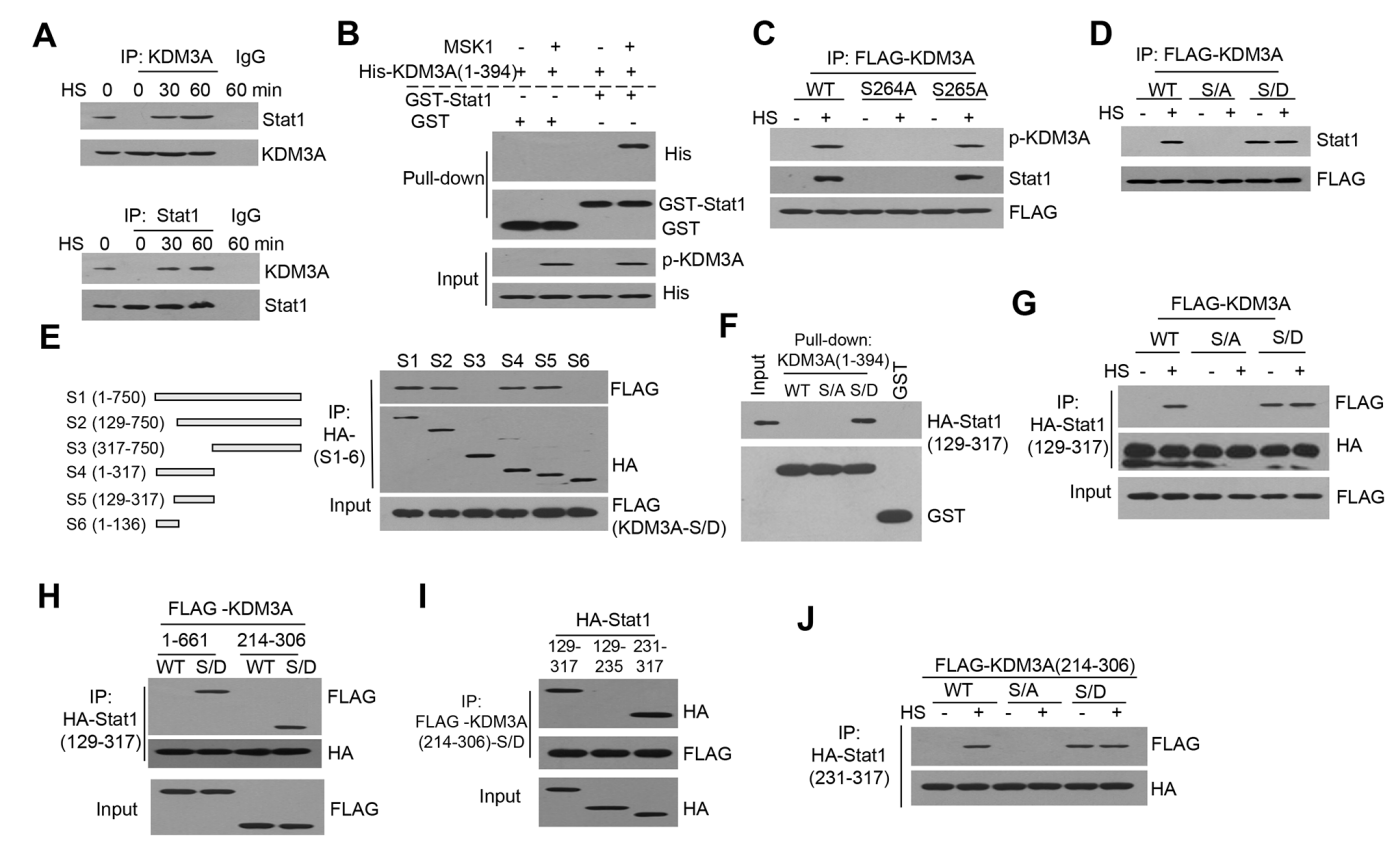
Phosphorylation is a prerequisite for the interaction between KDM3A and Stat1. (**A**) Co-IP assay of KDM3A and Stat1 in Jurkat cells. WCE samples were immunoprecipitated using an antibody for KDM3A (top) or Stat1 (bottom) and subjected to western blot using an antibody for Stat1 or KDM3A, respectively. IgG was used as a control. (**B**) p-KDM3A interacts with Stat1 in vitro. Recombinant MSK1 and His-KDM3A (1-394) were initially mixed for the kinase assay and were subsequently pulled down using GST or GST-Stat1. Western blot was performed using anti-His for p-KDM3A and anti-GST for both GST-Stat1 and the control, with p-KDM3A and His as inputs. (**C**) Co-IP assay to determine whether KDM3A phosphorylation at S264 is required for the interaction between KDM3A and Stat1. The cells were transfected with FLAG-tagged wild-type or S264A or S265A mutant KDM3A. The annotations are the same as those described in Fig. 1C. (**D**) Co-IP assay of wild-type (WT) and S264A(S/A) and S264D(S/D) mutant KDM3A to determine its ability to bind to Stat1 under HS. The WT-KDM3A, KDM3A-S264A and KDM3A-S264D constructs were individually transfected into Jurkat cells. Co-IP was performed as described in C. (**E**) Schematic presentation of the protein fragments (the digits in the parenthesis denote the first and last amino acids of the fragment) of Stat1 encoded by the of the wild-type (S1) and mutant (S2-S6) expression constructs (left panel). For the co-IP assay, a FLAG-KDM3A-S264D construct was co-transfected with each of the six HA-tagged Stat1 constructs. The HA antibody was used to immunoprecipitate KDM3A-S264D. (**F**) GST pull-down assays of the interaction between the recombinant 1-394 fragments of wild-type or S/A or S/D mutant KDM3A and the HA-Stat1(129-317) mutant. (**G**) Co-IP assay of the interaction between wild-type and S/A and S/D mutants of FLAG-KDM3A and the HA-tagged S5(129-317) mutant in Jurkat cells treated with (+) or without (-) HS. (**H**) Co-IP assay of the wild-type and S/D mutant FLAG-KDM3A fragment constructs covering residues 1-661 and 214-306 respectively, were co-transfected with S5 into Jurkat cells. WCE that were immunoprecipitated using the anti-HA antibody, which detects S5, were subjected to western blot using an anti-FLAG antibody. (**I**) Co-IP assay of three HA-Stat1 fragments, namely 129-317 aa (S5), 129-235 aa, and 231-317 aa, and the FLAG-tagged KDM3A-S264D (214-306) fragment. Following co-transfection with one HA-Stat1 fragment and the FLAG-tagged KDM3A-S264D (214-306) fragment, the WCE were immunoprecipitated using a FLAG antibody to pull down the 214-306 aa fragment of KDM3A-S264D in Jurkat cells. The HA-Stat1 fragments were detected via western blot for HA. (**J**) Co-IP assay of the wild-type and S/A and S/D mutants of the FLAG-tagged 214-306 fragment of KDM3A, which directly interacts with residues 231-317 of the HA-tagged Stat1 mutant. The annotations are the same as those described in G.

To determine the interaction between p-KDM3A and Stat1, we used antibodies targeting each protein to immunoprecipitate (IP) cell extracts for co-IP assays. We demonstrated that KDM3A and Stat1 interacted with one another only under HS (Fig. 3A). Based on a GST pull-down assay, MSK1 initially bound and phosphorylated KDM3A in vitro, but only p-KDM3A interacted with GST-Stat1 (Fig. 3B). By introducing S/A point mutations into KDM3A, we demonstrated that KDM3A-S264A, but not KDM3A-S265A, lacked this binding between KDM3A and Stat1 under HS (Fig. 3C), indicating that phosphorylation of KDM3A at S264 is critical for Stat1 binding. Next, we mutated S264 of KDM3A to aspartate (S/D) to mimic the phosphorylation of KDM3A at S264 in these cells. KDM3A-S/D co-immunoprecipitated with Stat1 even without HS (Fig. 3D), suggesting that although HS induces phosphorylation of both the Y701 and S727 residues of Stat1 [28], this phosphorylation was not required for Stat1 to interact with either p-KDM3A or KDM3A-S264D.

Then, we determined which region of Stat1 is required for its interaction with KDM3A-S264D in these cells. Among the Stat1 fragments S1, S2, S4, and S5 that interacted with KDM3A-S/D (Fig. 3E, top of right panel), the fragment S5 (residues 129-317, left panel) were the least required for this interaction. Based on GST pull-down assays, only the recombinant 1-394 fragment of KDM3A in its S264D form pulled down S5-Stat1 (Fig. 3F). Based on co-IP assays, HA-tagged Stat1 (129-317) interacted with full length S/D-KDM3A (Fig. 3G) and the shorter fragment S/D-KDM3A (214-306) (Fig. 3H), indicating that this 93-aa fragment of KDM3A interacts with Stat1. By performing another co-IP using an antibody against FLAG to detect FLAG-tagged KDM3A (214-306), we identified the 231-317 aa fragment of Stat1 was co-precipitated (Fig. 3I); this interaction between S264D-KDM3A (214-306) and Stat1 (231-317) was further confirmed in Fig. 3J.

Data from Fig. 1 and Fig. 3 revealed that p-MSK1 only interacted with p-KDM3A under HS, and p-KDM3A interacted with Stat1 even in its non-phosphorylated form. To address the detail correlations of MSK1, KDM3A, and Stat1 in heat-shocked cells, we further showed that p-MSK1 can be co-precipitated by a 214/306aa fragment of KDM3A under HS, suggesting a likely kinase versus substrate interaction for the phosphorylation of KDM3A at S264 (S7A Figure). Furthermore, the interaction of Stat1 and p-KDM3A was enhanced by extended incubation under HS, but not the interaction with p-MSK1 in the same cells and was not in the least enhanced (S7B Figure). However, the fact that the 93aa fragment of p-KDM3A could be co-precipitated by a 213/317aa fragment of Stat1 under HS indicates that the phosphorylated Y701 and S727 of Stat1 were not required for its interaction with p-KDM3A (Fig. 3J). Taken together, these results suggest these three factors do not exist in a complex, but sequentially take parts in the two functional stages: (1) activated MSK1 interacts and phosphorylates KDM3A-S264 under HS and (2) the recruitment of p-KDM3A via Stat1 to the promoter of target gene for HS inducing activation.

### p-KDM3A Mediates Chromatin Remodeling and Activates hsp90α

**Figure 4:**
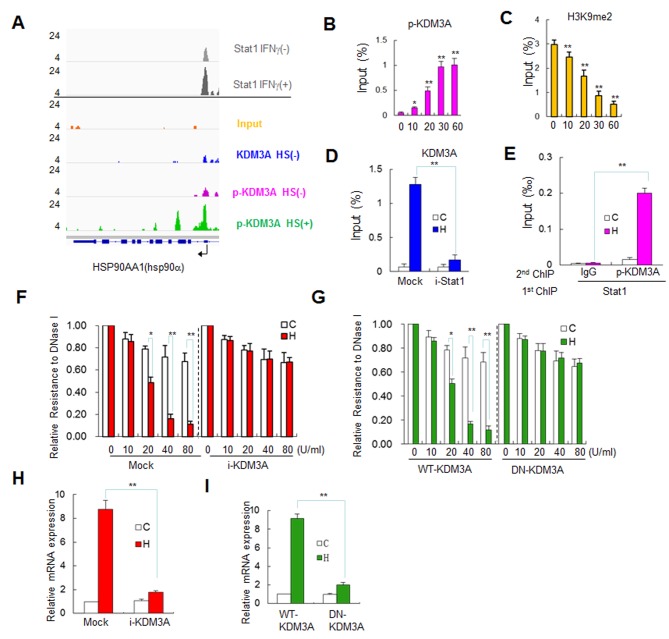
p-KDM3A is recruited by Stat1 to elicit chromatin remodeling of a Stat1 target gene. (**A**) Representative ChIP-seq tracks for KDM3A and p-KDM3A at *HSP90AA1 (hsp90α)* in Jurkat cells treated with or without HS. The annotations are the same as those in Fig. 2F. (**B and C**) The ChIP assay demonstrated the recruitment of p-KDM3A and H3K9me2 to the upstream region of human *hsp90α* upon HS treatment. The chromatin fragments were pulled down using and antibody against p-KDM3A (B) or H3K9me2 (C). The duration of HS treatment is shown (0–60 min). Each bar represents an average of at least three independent experiments, and the values are expressed as the means ± SD. The input percentage was detected via qPCR analysis for *hsp90α.* (**D**) ChIP assay showing the effects of either Stat1 (i-Stat1) or GFP shRNA (Mock) on the occupancy of KDM3A upstream of the corresponding gene in Jurkat cells. Each group of cells was divided into two groups, which were either subjected to HS (filled bars) or not (open bars). The chromatin fragments were pulled down using an antibody against KDM3A. (**E**) ChIP-reChIP assay showing that the recruitment of p-KDM3A to the upstream region of *hsp90α* is Stat1-dependent. The cells were transfected with FLAG-Stat1, and anti-FLAG was used during the initial ChIP to recover the Stat1-associated chromatin fragments. Then, these fragments were subjected to reChIP at each of the previous treatment temperatures using an antibody against p-KDM3A. IgG was used as a ChIP control. The qPCR data are expressed as described in D. (**F and G**) DNase I sensitivity analysis showing chromatin remodeling of the upstream region of *hsp90α* The cells that were transfected with either GFP (Mock) or KDM3A shRNA (i-KDM3A) (F) or the wild-type or D/N-KDM3A construct (G) were treated with HS (filled bars) or not (open bars). The nuclei were isolated and digested with DNase I as indicated, followed by genomic DNA extraction. The data are shown as the relative resistance to DNase I digestion normalized to non-DNase I treatment. The final concentration of DNase I is expressed in U/ml. (**H and I**) The mRNA expression level of *hsp90α* was determined via RT-qPCR analysis using GAPDH as a control in the cells treated with or without HS as described in F and G, respectively. Data are mean ± SD (*p<0.05, **p<0.01). The data used to make this figure can be found in S1 Data.

Next, we analyzed the MetaGene profile of p-KDM3A at the gene locus encoding *hsp90α* (hsp90aa1) under HS, which indicated the reads were enriched around the TSS of a cluster 1 gene. p-KDM3A under HS was markedly enriched at the TSS that is dominant over either non-heat shock p-KDM3A or non-phosphorylated KDM3A without HS (Fig. 4A). Interestingly, the p-KDM3A-enriched TSS region coincidently displays IFNγ-induced Stat1 binding at the *hsp90α* gene locus in HeLa S3 cells (Fig. 4A, top panel) according to Robertson et al [27]. Therefore, *hsp90α* is appropriately selected as a representative gene to further evaluate the mechanism underlying the targeting and functions of p-KDM3A in the human genome.

ChIP assays were then performed to examine the occupancy of p-KDM3A in the upstream sequences, its impact on the H3K9me2 level and in chromatin remodeling of *hsp90α*. We demonstrated that p-KDM3A was gradually enriched near the GAS element of *hsp90α* over time under HS (Fig. 4B), while the level of endogenous H3K9me2 decreased (Fig. 4C). This result suggests that p-KDM3A is directly involved in the demethylation of H3K9me2. Interestingly, once Stat1 was knocked down using a specific shRNA, the heat-shock-induced occupancy of p-KDM3A was abrogated in these cells (Fig. 4D), moreover, KDM3A-S/D mimic was no longer occupied even without HS (S8 Figure). In contrast, Stat1 binding remained following KDM3A knockdown (S9C Figure). ChIP/reChIP assays also demonstrated that p-KDM3A occupancy at the GAS element is Stat1-dependent (Fig. 4E). For DNase I hypersensitivity analysis, we set the sensitivity level without DNase I to 1.00 on the *y*-axis, representing a 100% “resistance” to this enzyme. As the amount of DNase I increased, the resistance to DNase I digestion significantly decreased in the upstream region of *hsp90α* in mock shRNA-transfected cells under HS (Fig. 4F, filled bars in left panel). In contrast, the HS-mediated changes in DNase I sensitivity at the GAS element were absent from KDM3A shRNA-transfected cells (Fig. 4F, right panel). Furthermore, in non-functional KDM3A H1120Y mutant (DN-KDM3A)-transfected cells [10], a similar profile lacking any clear changes in HS-dependent DNase I sensitivity was found (Fig. 4G). These data indicate that HS-mediated DNase I sensitivity at the GAS element is dependent on KDM3A demethylase activity. The HS-induced activation of *hsp90α*, as revealed by RT-qPCR analysis of its mRNA expression, was markedly reduced in KDM3A-knockdown cells (Fig. 4H) and in DN-KDM3A-transfected cells (Fig. 4I).

### MSK1 Is Indispensable for the Occupancy of KDM3A, Chromatin Remodeling, and Gene Activation upon Heat Shock

**Figure 5:**
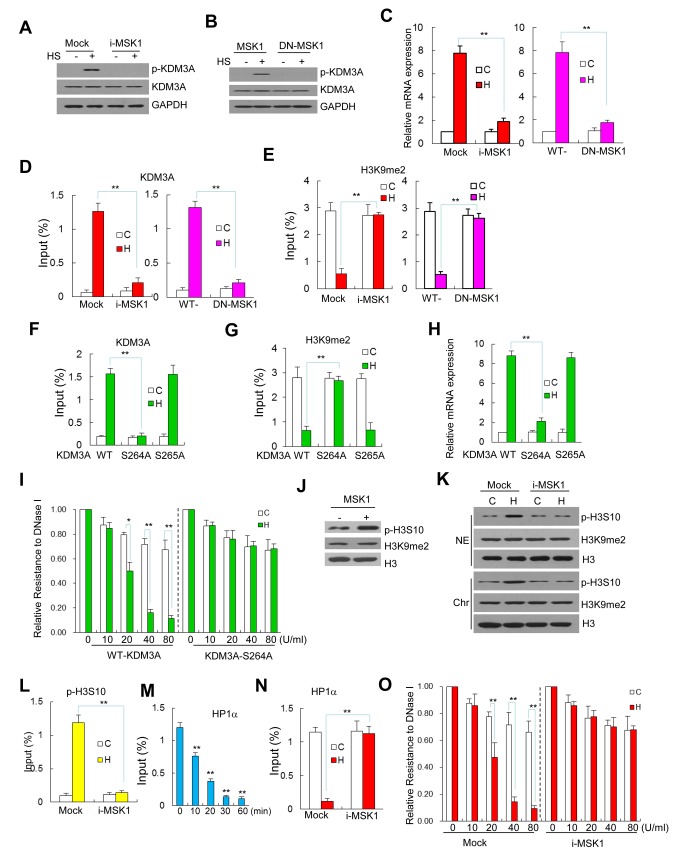
MSK1 is a prerequisite for Stat1 target gene activation via KDM3A phosphorylation. (**A and B**) The phosphorylation of KDM3A was abolished in (A) MSK shRNA (i-MSK1) and (B) the DN-MSK1-transfected cells subjected to HS (+) compared to the control GFP shRNA-transfected cells. (**C**) The mRNA expression level of hsp90α was severely impaired in the heat-shocked cells that were transfected with either MSK1 shRNA (i-MSK, left) or DN-MSK1 (right). (**D and E**) The occupancies of KDM3A (D) and H3K9me2 (E) upstream of *hsp90α* under HS in i-MSK1- (left) and DN-MSK1-transfected cells (right). (**F–H**) The wild-type and S264A KDM3A constructs were transfected into Jurkat cells as described above; KDM3A-S265A was transfected as a non-functional control that displays similar effects to transfection with wild-type KDM3A. The HS-induced input percentage of KDM3A was eliminated (F); that of H3K9me2 was retained at a high level (G), and the HS–induced mRNA expression levels were significantly reduced in the KDM3A-S264A mutant-transfected cells under HS (H) but not in the wild-type or S265A KDM3A-transfected control cells. (**I**) The cells that were transfected with wild-type KDM3A or KDM3A-S264A were treated with HS (filled bars) or not (open bars). DNase I sensitivity analysis showing chromatin remodeling upstream of *hsp90α*. The annotations are the same as those in Fig. 4F. (**J**) H3S10 phosphorylation assay in vitro. Recombinant MSK1 was incubated for 30 min in histones extracted from HeLa cells. Then, the reaction mixtures were separated via SDS-PAGE. Western blot was performed using antibodies against pH3S10, H3K9me2, and H3. (**K**) MSK1 phosphorylates H3S10 in Jurkat cells under HS. Jurkat cells were transfected with GFP (Mock) or MSK1 shRNA and then subjected to HS for 60 min. The nucleoplasmic protein (NE) and chromatin fractions (Chr) were extracted for western blot using antibodies against pH3S10, H3K9me2, and H3. (**L**) The effect of MSK1 on H3S10 occupancy at the GAS of *hsp90α* under HS. The cells were treated as described in K. ChIP assays were performed using an antibody for pH3S10. The input percentage was determined via qPCR analysis for *hsp90α*. (**M**) A ChIP assay demonstrated the recruitment of HP1*α* upstream of human *hsp90α* upon HS treatment. The chromatin fragments were pulled down using a specific antibody against HP1*α*. The duration of HS treatment is shown (0–60 min). Each bar represents an average of at least three independent experiments, and the values are expressed as the means ± SD. The input percentage was determined via qPCR for *hsp90α* (**N**) The effect of MSK1 on the recruitment of HP1*α* to the GAS of *hsp90α* under HS. Jurkat cells that were transfected with GFP (Mock) or MSK1 shRNA were subjected to HS for 60 min. A ChIP assay was performed as described in M. (**O**) DNase I sensitivity analysis of chromatin remodeling upstream of *hsp90α*. The cells that were transfected with GFP (Mock) or MSK1 shRNA (i-MSK1) were treated with HS (filled bars) or not (open bars). The annotations are the same as those described in Fig. 4F. Data are mean ± SD (*p<0.05, **p<0.01). The data used to make this figure can be found in S1 Data.

Jil1, the *Drosophila* ortholog of human MSK1, is activated in response to heat shock [20] and phosphorylates H3 to elicit chromatin relaxation, facilitating the binding of additional regulatory proteins [21]. In this study, we demonstrated that MSK1 is also activated in heat-shocked cells, as shown in Fig. 1E. To further address the detailed functions of MSK1 in KDM3A, we transfected the cells with either shRNA (i-MSK1) or a dominant negative (DN) mutant of MSK1; the phosphorylation of KDM3A at S264 under HS was blocked in these cells compared to the wild-type control cells (Fig. 5A and 5B and S10A–C Figure). However, similar to KDM3A knockdown, MSK1 knockdown did not affect the occupancy of Stat1 upstream of *hsp90α* (S10D Figure). i-MSK1 and DN-MSK1 also significantly impaired the mRNA expression of *hap90α* under HS (Fig. 5C), similar to the results using i-KDM3A and DN-KDM3A (Fig. 4H and 4I). These results indicate that MSK1 is the critical kinase that is responsible for the phosphorylation of KDM3A at S264 under HS. Then, we demonstrated that these reduced expression profiles in the presence of i-MSK1 and DN-MSK1 were based on a change in the occupancy of KDM3A at the GAS of *hsp90α* (Fig. 5D); a high expression level of H3K9me2 was detected (Fig. 5E). Furthermore, using the S264A mutant of KDM3A, the MSK1-mediated occupancy of KDM3A at the GAS was abolished (Fig. 5F), the expression levels of H3K9me2 remained elevated (Fig. 5G), and HS-induced mRNA gene expression was markedly reduced (Fig. 5H). In contrast, using the S265A mutant of KDM3A, identical results were obtained compared to wild-type KDM3A, as shown in the respective figures. Additionally, the importance of residue S264 of KDM3A was further demonstrated in KDM3A-S264A-transfected cells, which exhibited strongly reduced HS-induced DNase I hypersensitivity at the GAS region of *hsp90α* (Fig. 5I). It is, therefore, notable that the occupancy of p-KDM3A at GAS is required for KDM3A to display its demethylase activity on H3K9me2 and elicit chromatin remodeling at the GAS to activate the *hsp90α* gene.

MSK1 is a major kinase responsible for the phosphorylation of histone H3, including at S10 and S28 [29], and the phosphorylation of H3S10 facilitates the accessibility and transcriptional competence of a specific chromatin region in the genome [18],[30],[31]. Next, we demonstrated via western blot that the expression of phosphorylated H3S10 (p-H3S10) increased in heat-shocked Jurkat cells and was inhibited by transfection with specific MSK1 shRNA (Fig. 5J and 5K). A ChIP assay also verified the inhibitory effect of this shRNA on the occupancy of p-H3S10 at the GAS region under HS (Fig. 5L). In addition, the ChIP assay revealed that HP1α, the only HP1 isoform in the GAS region of *hsp90α*, is expressed at high levels preceding HS and reduced rapidly to minimal level within the first 30 min of HS treatment in Jurkat cells (Fig. 5M and 5N). Because the expression of p-H3S10 at the GAS was accompanied by an increase in acetylation of H3K9 but not H3K14 upon HS treatment [28], the phosphorylation of H3S10 by MSK1 may provide an open chromatin structure to recruit p-KDM3A via Stat1, thus facilitating the binding of additional regulatory proteins. This explained why the HS-induced DNase I hypersensitivity was severely impaired by the knockdown of MSK1 (Fig. 5O). Although the outcome elicited by MSK1 was similar with that of the KDM3A-S264A transfected (Fig. 5I), it may indicate that a novel aspect of MSK1 functioned on human chromatin remodeling under heat shock.

### The Phosphorylation of KDM3A Determines the Differential Expression of Stat1-Targeted Genes under Cellular Stress Conditions

**Figure 6:**
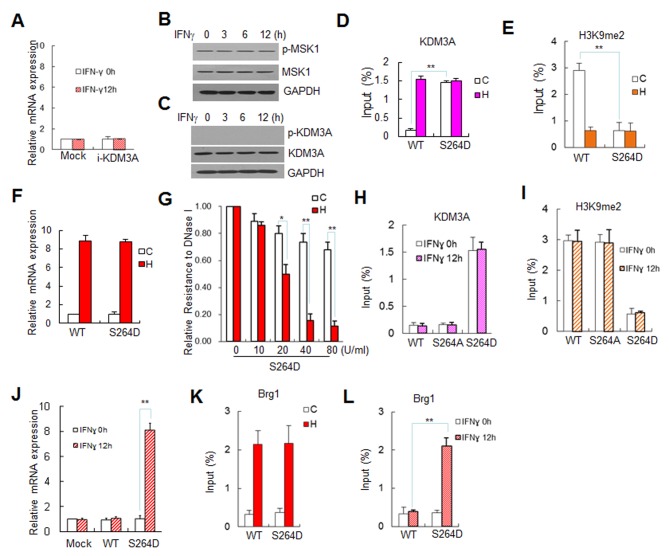
p-KDM3A regulates the expression of *hsp90α* under HS or IFN-γ treatment. (**A**) The effects of KDM3A on the mRNA expression levels of *hsp90α* in Jurkat cells under IFN-γ treatment. The cells were transfected with GFP (Mock) or KDM3A shRNA. The mRNA expression level was determined via RT-qPCR (IFN-γ: slanted line-filled bars; control: open bars). Other details are the same as those described in Fig. 4I. (**B**) Western blot of phosphorylated MSK1 (p-MSK1) in Jurkat cells that were treated with IFN-γ for 3, 6, or 12 hr. The p-MSK1 levels remained unchanged during IFN-γ treatment. The MSK1 and GAPDH antibodies were used as positive and loading controls, respectively. (**C**) Western blot of p-KDM3A, which was not detected in the IFN-γ-treated cells, although the non-phosphorylated KDM3A expression level remained unchanged. The antibodies against p-KDM3A, KDM3A, and GAPDH were used as described in B. (**D-F**) The effect of KDM3A-S264D on the recruitment of KDM3A and the H3K9me2 level at the GAS of *hsp90α* compared to that of wild-type KDM3A under HS. The Jurkat cells were transfected with wild-type KDM3A or KDM3A-S264D. ChIP assays were performed using an antibody for FLAG (D) or H3K9me2 (E), and the mRNA expression levels were determined via RT-qPCR (F). (**G**) The cells were transfected with KDM3A-S264D and then treated with HS (filled bars) or not (open bars). DNase I sensitivity analysis showing chromatin remodeling upstream of *hsp90α*. The annotations are the same as those in Fig. 4F. (**H–J**) The effects of IFN-γ treatment on the recruitment of KDM3A (H) and H3K9me2 (I) to *hsp90α* and the mRNA expression level of *hsp90α* (J) in cells that were transfected with KDM3A-S264D compared to those transfected with wild-type or S/A-mutant KDM3A. (**K and L**) The effects of KDM3A-S264D (a p-KDM3A-S264 mimic) on Brg1 recruitment at *hsp90α* under HS and IFN-γ treatment. Jurkat cells were transfected with either wild-type KDM3A or KDM3A-S264D and then treated with HS for 60 min (K) or IFN-γ for 12 hr (L). Data are mean ± SD (*p<0.05, **p<0.01). The data used to make this figure can be found in S1 Data.

We previously reported that in contrast to HS treatment, IFN-γ treatment does not induce the expression of *hsp90α* or other related genes, such as *CIITA-*pIV, in Jurkat cells [28]. In this study, we demonstrated that p-KDM3A occupied at the GAS region of *hsp90α* (Fig. 4B), and its expression is efficiently induced under HS (Fig. 4H and 4I). IFN-γ did not induce the mRNA expression of this gene, independent of the presence of KDM3A in these cells (Fig. 6A). Unlike HS treatment, as shown in Fig. 1D and 1E, IFN-γ treatment did not induce the expression of MSK1 or activate the kinase activity of MSK1 (Fig. 6B), thus preventing the specific phosphorylation of KDM3A at S264 in IFN-γ-treated cells (Fig. 6C). These data indicate that only HS treatment activates MSK1 to phosphorylate KDM3A at S264, but this pathway is not activated in IFN-γ–treated cells. Therefore, we conclude that the expression level of p-KDM3A is the critical difference between the impact of HS and IFN-γ on the activation of their target genes in Jurkat cells.

To determine the mechanism by which p-KDM3A differentially functions in cells under different treatments, we transfected the cells with mutant KDM3A-S264D to mimic the phosphorylation of the critical S264 of KDM3A. We demonstrated that KDM3A-S264D occupied the GAS element of *hsp90α* either with or without HS treatment (Fig. 6D) and strongly reduced the H3K9me2 expression to the basal level (Fig. 6E). In contrast, *hsp90α*mRNA expression and DNase I hypersensitivity for the KDM3A-S264D mutant were similar to those for the wild-type enzyme under HS but not the control conditions (Fig. 6F and 6G).

Then, the aforementioned transfected cells were treated with IFNγ. The ectopically expressed KDM3A-S264D was efficiently recruited to the GAS region of *hsp90α* and the expression level of H3K9me2 was markedly reduced in the presence or absence of IFN-γ. However, wild-type and S264A mutant KDM3A did not bind to the GAS in IFNγ-treated cells and did not display any demethylase activity on H3K9me2 (Fig. 6H and 6I). Notably, KDM3A-S264D, but not the wild-type or S/A mutant counterparts, rendered *hsp90α* to be susceptible to IFN-γ treatment, as that shown under HS (Fig. 6J, slanted line-filled bars compared to the open bars).

**Figure 7:**
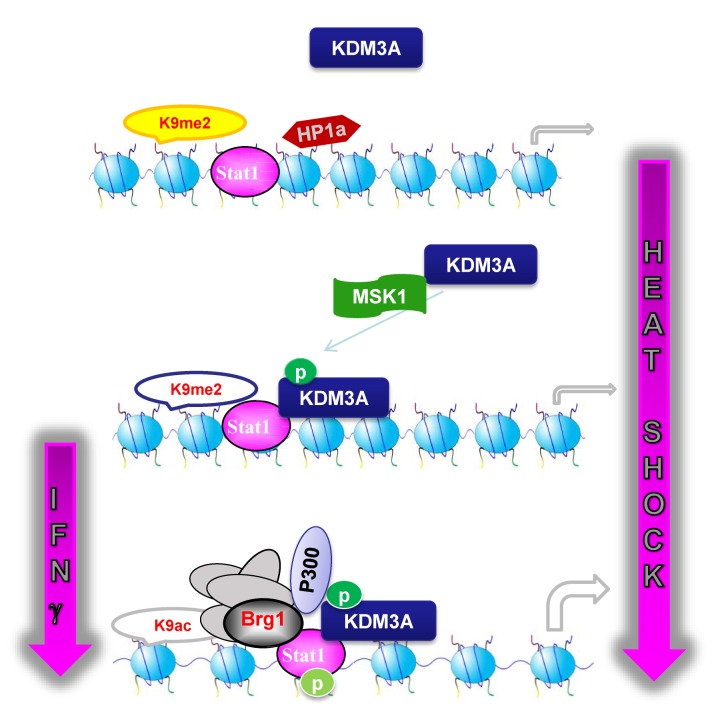
Schematic of a two-step model of HS-induced gene activation via the MSK1-p-KDM3A-Stat1 pathway.

The above results indicate that in untreated Jurkat cells, the ectopic KDM3A S/D mutant occupied the GAS and decreased the H3K9me2 level, but for an unknown reason, *hsp90α*mRNA expression was not induced. Therefore, we transfected wild-type and S/D mutant KDM3A into Jurkat cells to examine the occupancy of the Brg1 chromatin remodeling complex at the GAS before and after HS treatment or after IFNγ treatment. The ChIP data indicated that only when KDM3A-S/D was transfected did Brg1 efficiently occupy the GAS following both HS (Fig. 6K) and IFNγ treatment (Fig. 6L), but this binding was never constitutive at the GAS. However, transfected KDM3A and its S/A, S/D mutants did not affect Stat1 binding at the GAS (S11 Figure). This result agrees with our previous report that Brg1 is only recruited by p-Stat1 that is induced in response to HS treatment [28]. In IFNγ-treated cells, p-Stat1 also occupied the GAS [32], possibly providing a docking site for KDM3A-S/D and activating *hsp90α*. Therefore, it is conceivable that Stat1-mediated p-KDM3A recruitment is necessary but not sufficient for gene activation (Fig. 7). Our data indicate that the level of gene activation under HS or IFN-γ treatment is determined by the potential for an external stimulus to activate MSK1, which phosphorylates KDM3A. The two-step model in Fig. 7 shows that, first, MSK1-phosphorylated KDM3A is recruited by Stat1 to remove the repressive mark H3K9me2, and second, p-Stat1 mediates Brg1 complex recruitment to fully activate the target gene.

## Discussion

KDM3A is the second identified JmjC domain lysine demethylase (JHDM2A) that is specific for the demethylation of H3K9me2/me1. This demethylase contains a JmjC domain at 1058-1281 aa and a zinc finger domain at 662-687 aa [10]. Although certain TFs can induce KDM3A expression [13],[33]–[35] or interact with KDM3A [11],[14],[36], our understanding of the relationship between its modification and function has not been fully elucidated since its discovery.

In this study, we demonstrate that KDM3A is phosphorylated at S264 by MSK1 under heat shock. Specifically, S264 of KDM3A is approximately 400 residues from the N-terminus of the zinc finger domain, which performs no known function [10]. We then perform ChIP-Seq analysis to determine the genome-wide distribution of HS-induced p-KDM3A in Jurkat cells. To our surprise, ChIP-Seq data have shown that either with or without HS, the peaks of p-KDM3A could occupy the mappable genome at a comparable percentage. We then analyze the MetaGene profiles of p-KDM3A under HS, which shows the reads are enriched around the TSS at all of the five gene loci encoding the *hsp90α* (Fig. 4A) and the other genes (Fig. 2F); while those of the constitutive p-KDM3A only show much lower or minimal occupancy at these loci (the fourth versus the third rows in the bottom panels of Fig. 4 and 2). This finding suggests the p-KDM3As, either induced under stress (HS) or expressed in the normal life cycle of the cells, are functionally diverse through distribution to each distinct gene locus in the genome. In addition, the occupancy of p-KDM3A on Myo7B-Lims2 site is reduced under HS. The p-KDM3A in non-HS cells is likely phosphorylated by other kinase(s) or even the constitutively expressed MSK1 (Fig. 1E). These kinases can be activated via specific signaling pathway(s), such as IFNα [21], and exhibit their own function(s) on the specific constitutively expressed genes in the cells.

The TF motifs from ChIP-Seq data indicate that the p-KDM3A-bound sites are similar to those of some TFs, including Stat1. The phosphorylation of S264-KDM3A is a prerequisite for its efficient interaction with the TF Stat1, and residues 231-317 in the coiled-coil domain of Stat1 interact with the p-KDM3A in vitro. We suggest that this Stat1/p-KDM3A interaction represents a TF that directs KDM3A to an appropriate upstream element of its target gene to demethylate H3K9me2.

Because MSK1 is activated in response to a vast array of environmental stress stimuli via the p38 or ERK pathway to phosphorylate histone and HMG proteins [37],[38], MSK1 is involved in chromatin remodeling [21],[39]. We demonstrate that MSK1 is activated by HS but not IFNγ treatment and that p-KDM3A efficiently reduces the level of H3K9me2 at the GAS of *hsp90α* and renders this region susceptible to DNase I treatment.

Our data suggest that the p-KDM3A-mediated reduction in H3K9me2 expression is a major step of gene activation in Jurkat cells. Because no gene expresses efficiently in the presence of high level of H3K9me2 in Jurkat and Raji cells in response to either HS or IFNγ treatment (S12 Figure and ref. [28]). Hence the outcome of gene activation under HS or IFN-γ treatment is determined by the potential for the stimulus to activate MSK1 to phosphorylate KDM3A.

KDM3A-S264D was used in this study to mimic the function of phosphorylated KDM3A-S264 in vivo. We demonstrate that this S264D mutant directly interacts with Stat1 to occupy the GAS element regardless of heat shock. Although the KDM3A-S264D mutant constitutively binds to the GAS element, H3K9me2 remains at a basal level under IFN-γ treatment, similar to the results under HS treatment; in contrast, non-phosphorylated KDM3A does not interact with Stat1, is not recruited to the GAS element, and does not reduce the level of H3K9me2 when exposed to IFN-γ.

H1120 in the JmjC domain is indispensable for the demethylase activity of KDM3A [10]. However, the phosphorylation of KDM3A-S264 exerts the same effects, including H3K9me2 reduction and DNase I hypersensitivity at Stat1 target genes. Therefore, it is logical to propose that the Stat1-mediated recruitment of the p-KDM3A represents a specific pathway by which the demethylase activity of KDM3A is regulated under heat shock.

In summary, heat shock is a physical stimulus that broadly affects the expression of a variety of genes in human cells, likely in a general manner. In addition to the activation of the well-accepted heat shock factor and heat shock element (HSF/HSE) pathways to induce expression of heat-shock-related genes, we present a novel, generalized heat-shock-induced activation mechanism that is centered on the phosphorylation of KDM3A. (1) p-KDM3A-S264 is enriched genome-wide at the promoter region of several genes, including heat-shock-related genes, under heat shock; (2) p-KDM3A is guided by a TF to the binding element of TF in the genome; (3) the genomic occupancy of p-KDM3A at its target genes is a prerequisite for the demethylase activity of KDM3A in situ; and (4) the phosphorylation of KDM3A is specifically dependent on the upstream stimulus-dependent kinase activity of MSK1 in HS- but not IFN-γ-treated Jurkat cells.

## Materials and Methods

### Antibodies

Antibodies against KDM3A, p-MSK1, GAPDH, H3K9me2, and H3K9me3 and recombinant activated MSK1 were purchased from Millipore Biotech (Billerica, MA, United States). The FLAG and M2 antibodies were purchased from Sigma. The GST, MSK1, MSK2, HA, and Stat1 antibodies were purchased from Santa Cruz Biotechnologies (Santa Cruz, CA, US). The anti-phosphorylated serine (p-Ser) (antibody catalog number AB1603) was purchased from Merck (Darmstadt, Germany). A specific antibody against p-S264-KDM3A was produced by Beijing B&M Biotech (Beijing, China) using the synthesized peptide VKRKSSENNG, corresponding to residues 260–269 of KDM3A, as an antigen.

### Plasmids

The FLAG-tagged MSK1 eukaryotic expression plasmid was constructed by cloning MSK1 into the pcDNA6-FLAG vector using a PCR product from a Jurkat cell cDNA library. We inserted point mutations at amino acids 165 (D to A) and 565 (D to A) in full-length FLAG-MSK1 to produce DN-MSK1 [40]. The FLAG-tagged KDM3A eukaryotic expression plasmid was a gift from Dr. Zhong-Zhou Chen of China Agricultural University. We inserted a point mutation at amino acid 1120 (H to Y) to produce DN-KDM3A [10], and we generated five individual point mutants of KDM3A: S264A, S265A, S445A, S463A, and S264D. The KDM3A fragment from 214-306 was subcloned using the PCR product of full-length FLAG-KDM3A. The MSK1 and KDM3A shRNA oligonucleotide sequences were designed by OriGene Technologies, Inc. (Rockville, MD, USA) and inserted into the HindIII/BamHI site of the pRS vector. shRNA-Stat1 was purchased from OriGene Technologies, Inc. The truncation mutants of Stat1 (S2 and S4-S6) were described previously [28]. A new construct of S3 (317–750 aa) was subcloned using the PCR product of full-length HA-Stat1 (S1). We constructed Stat1 (129–235) and Stat1 (231–317). The primers that were used to generate the MSK1, KDM3A, and Stat1 mutant plasmids are listed in S5 Table.

### RT-qPCR

RT-qPCR was performed as described previously [41],[42]. The relative expression levels of DNAJB1, SERPINH1, SMIM20, RNASEK, and HSP90AA1 (*hsp90α*) were normalized to those of GAPDH using the comparative CT method according to the manufacturer's instructions (Rotor-Gene RG-3000A Real-Time PCR System, Corbett Research, Australia). The specific primers corresponding to the above genes are listed in S6 Table. The experiments were repeated at least three times, and statistical analysis was performed on the individual experimental sets. All of the values in the experiments are expressed as the means ± SD.

### ChIP-qPCR Assays

The ChIP assays were performed as described previously [41],[42]. The primers used for DNAJB1, SERPINH1, SMIM20, RNASEK, and HSP90AA1 (*hsp90α*) are listed in S7 Table. The percentage of ChIP DNA relative to the input was calculated and expressed as the mean ± SD of three independent experiments [43].

For ChIP-reChIP analysis [28], first, Jurkat cells were transiently transfected with FLAG-tagged Stat1 expression plasmids prior to further treatment. The chromatin fragments from the sonicated cells with or without HS treatment were used as the input, which was then immunoprecipitated using an anti-Flag M2 affinity gel (F1). Aliquots of the F1 chromatin fragments were reverse cross-linked to obtain DNA for qPCR assays or were saved for re-IP using an antibody against KDM3A or p-KDM3A for reChIP assays (F2). The DNA that was extracted from the chromatin fragments subjected to reChIP was re-amplified using the primer sets used for qPCR. The amount of KDM3A or p-KDM3A that was recruited by the antibody against Stat1 at 42°C was quantified relative to that recruited at 37°C, which was normalized to 1.

### ChIP DNA Preparation for High-Throughput Sequencing

For ChIP-Seq, the chromatin fragments of 1×10^7^ Jurkat cells with or without HS treatment were immunoprecipitated using IgG or an antibody against KDM3A or p-KDM3A. The DNA fragments were end-repaired, adenylated, ligated to adaptors, and PCR-amplified for 18 cycles. The PCR products corresponding to bp 250-450 were gel-purified, quantified and stored at −80°C until use for sequencing. For high-throughput sequencing, the libraries were prepared according to the manufacturer's instructions, and to the samples were analyzed using an Illumina GAIIx system for 80-nt single-end sequencing (ABLife, Wuhan, China).

### ChIP-seq Data Analysis

The data were analyzed using Active Motif; the flow chart of analysis is shown in S13 Figure. After removing the adaptors and low-quality bases, the reads (36 bp in length) were mapped to the human genome (hg19) using the BWA algorithm with the default settings. The clean reads that passed through the Illumina purity filter and aligned with less than two mismatches and without duplicates were saved as BED files for use in subsequent analyses. The mapped reads were inserted into seqMINER to obtain the Meta Gene distribution profile, and the genes were distributed into three clusters based on their distribution profiles. The reads files were converted to Wig files, which were inserted into the IGV 2.3 Genome Browser with the peak height set at 4–24 to determine the peak binding profiles.

For peak calling, the mapped BED files were inserted into SICER V1.1 [23] (estimated false discovery rate [FDR] threshold  = 1×10^−10^; window size: 200 bp; fragment size: 200 bp; gap size: 200 bp; hg19 genome database) and MACS 1.4.2 (*p*-value cutoff  = 1×10^−7^; tag size: 36 bp; band width: 150 bp; model fold = 8, 24) [44] using the pooled input (control/heat shock) and IgG experiment reads files as backgrounds. The NCBI Gene Expression Omnibus (http://www.ncbi.nlm.nih.gov/geo/) accession number for the ChIP-seq data is GSE62309.

The GO and MSigDB Pathway analyses were conducted using GREAT 2.02 on the SICER intervals data limited to the regulator regions (from −5 kb to approximately +2 kb of the TSS). The pathway analysis database in GREAT is the MSigDB from the Gene Set Enrichment Analysis. The binomial *p*-value reflects the significance of the targeted genes enriched in a GO term.

To identify the genome sites with more p-KDM3A after heat shock, we used the p-KDM3A HS (+) MACS interval peaks in Active Regions (in locations where only one sample had an interval, which defines the Active Region) to perform a sample comparison with peak metrics against the p-KDM3A HS (−). The unique intervals were annotated into genes (between 10 kb upstream and 10 kb downstream). The GO analysis of these genes was described above.

Transcription factor motifs were identified around p-KDM3A SICER islands (FA files) after heat shock using MEME (version 4.9.1) [45]. The database JASPAR_CORE_2014_vertebrates was used.

### DNase I Sensitivity Assay

Jurkat cells were transiently transfected with shRNA-MSK1 or shRNA-KDM3A. A total of 1×10^7^ cells were washed twice in PBS, and the nuclei were extracted as described above and digested with DNase I (ranging from 0 to 80 units/ml) on ice for 10 min. The DNase I digestion was terminated by incubating in stop buffer (Promega, M6101) at 65°C for 10 min. Then, the nuclei were digested with 50 µg/ml RNase A at 37°C for 60 min and 50 µg/ml proteinase K at 50°C overnight. The genomic DNA was purified via phenol/chloroform extraction and ethanol precipitation [46],[47]. Aliquots of 10 µg DNA were purified for qPCR using the primers described for the ChIP-qPCR assays.

### GST Pull-Down Assay

The GST-Stat1 fusion protein was expressed in *Escherichia coli* (BL21 DE3) and purified using glutathione-sepharose. GST and GST-Stat1 were bound to glutathione-sepharose, and 10 µl packed beads containing 5 µg the GST or GST-Stat1 fusion protein were incubated in the product of the kinase assay for MSK1 and KDM3A. After overnight incubation at 4°C, the beads were washed three times, and the bound proteins were analyzed via western blot.

### Co-IP and Immunoblot Analyses

The Co-IP analyses were performed using approximately 500 µg protein samples that were incubated in a specific antibody for 2 hr at 4°C. In total, 20 µl Protein A (or G)-agarose were added, and the samples were incubated at 4°C overnight. Then, the pellets were washed with RIPA buffer, followed by the addition of 40 µl 1× Laemmli buffer. Then, the samples were resuspended and boiled. The samples were separated via SDS-PAGE and analyzed via sequential western blot using individual antibodies [48].

### In Vitro Kinase Assay and Mass Spectrometry

Recombinant MSK1 (Millipore Biotech) was incubated in 1 µg purified wild-type or mutant KDM3A (1-394) in the presence of 50 µM ATP or 5 µCi [γ-^32^P]ATP in kinase buffer (10 mM Tris, pH 7.4; 10 mM MgCl2, 150 mM NaCl) for 30 min at 30°C. The reaction products were resolved via SDS–PAGE for western blot using specific antibodies; alternatively, the ^32^P-labeled proteins were visualized via autoradiography. Recombinant MSK1 was incubated in 1 µg of the synthesized peptide cVKRKSSENNG, corresponding to residues 260-269 of KDM3A, in the presence of 50 µM ATP in kinase buffer for 30 min at 30°C. The reaction products were purified for mass spectrometric analysis (Institute of Microbiology, CAS, China). Recombinant MSK1 was incubated in full-length GST-KDM3A for the kinase assay; then, 2 µg histone from HeLa cells was added to demethylation buffer (50 mM Tris, pH 8.0, 50 mM NaCl, 2 mM L-ascorbic acid, 1 mM α-ketoglutarate, 50 µM Fe(NH_4_)_2_(SO_4_)_2_) at 37°C for 2 hr, and the reaction was terminated by adding SDS-PAGE loading buffer. The results were analyzed via western blot using specific antibodies.

The numerical data in all figures are included in S1 Data.

## Supporting Information

S1 Data **The numerical data in all figures.**
(XLS)

S1 Figure **KDM3A is recruited to the upstream of** ***hsp90α*** **in response to heat shock.** The ChIP assay demonstrated the recruitment of KDM3A, KDM4A, and KDM4C upstream of human *hsp90α* upon HS treatment. The cells were transfected with FLAG-tagged KDM3A, KDM4A, or KDM4C. The chromatin fragments were pulled down using a specific antibody against FLAG. The duration of HS treatment is indicated at the bottom of each bar (0–60 min). The annotations are the same as those in Fig. 4B. Data are mean ± SD (*p<0.05, **p<0.01). The data used to make this figure can be found in S1 Data.
(TIF)

S2 Figure **Characterization of the antibody specific for p-KDM3A-S264.** (**A**) Western blot indicating the antibody efficiency for p-KDM3A using KDM3A phosphorylated by MSK1 in vitro. The phosphorylated peptide cVKRK(p)SSENNG (p-peptide) was used as a specific competitor, and the non-phosphorylated peptide was used as a control. (**B**) The cells were treated with HS for 0, 30, or 60 min. The specificity of the anti-p-KDM3A antibody was determined via western blot, as described above.
(TIF)

S3 Figure **p-KDM3A interacts with MSK1 in heat-shocked cells.** (**A**) The cells were transfected with FLAG-S/A-KDM3A. Co-IP assays were performed using an anti-FLAG antibody, followed by western blot using antibodies for p-MSK1 and FLAG. (**B**) The cells were transfected with FLAG-tagged wild-type or DN-MSK1. Co-IP was performed using an anti-FLAG antibody, followed by western blot using anti-KDM3A and anti-FLAG antibodies. The inputs and the IP using IgG are shown as controls.
(TIF)

S4 Figure **Histone H3K9me2 demethylation assay in vitro.** The histone demethylation assay demonstrated that the phosphorylation of KDM3A at S264 did not affect the demethylase activity of KDM3A on H3K9me2. Recombinant MSK1 and GST-KDM3A were initially mixed for the kinase assay and were subsequently added to histones that were purified from HeLa cells for the demethylase activity assay. The reaction products were separated via SDS-PAGE for western blot using the H3K9me2 antibody. Other antibodies used included those used for the kinase assay control: H3K9me3 as a demethylase activity control and MSK1, GST, and H3 as input controls.
(TIF)

S5 Figure **GO and pathway analyses of the KDM3A HS (-) and p-KDM3A HS (-) binding genes.**
(TIF)

S6 Figure **Motif analysis of the p-KDM3A-enriched regions using discriminative DNA motif discovery (DREME) [49].**
(TIF)

S7 Figure **Interaction between Stat1 and p-KDM3A.** (**A**) Jurkat cells were transfected with FLAG-KDM3A(1-661), FLAG-KDM3A(661-1321) and FLAG-KDM3A(214-306) and treated with HS for 1 hr. Co-IP assays were performed using an anti-FLAG antibody, followed by western blot using antibodies for p-MSK1, MSK1, and FLAG. (**B**) The cells were treated with HS for the indicated time (min). Then, the cell lysates were immunoprecipitated using an anti-Stat1 antibody, followed by western blot using antibodies against Stat1, MSK1, and p-KDM3A. The inputs and IP using IgG are shown as controls.
(TIF)

S8 Figure **The effects of Stat1 knockdown on the occupancy of phosphorylation mimic of KDM3A.** (**A**) The cell extracts from Jurkat cells transfected with either the i-Stat1 or mock vector were used for western blot. Based on western blot for Stat1, only a minimal level of Stat1 was detected in the i-Stat1-transfected cells. GAPDH was used as a control. (**B**) The Jurkat cells were co-transfected with KDM3A-S/D and Mock or i-Stat1. A ChIP assay showed the effect of knockdown of Stat1 on the occupancy of KDM3A-S/D at the upstream of *hsp90α*. Data are mean ± SD (**p<0.01). The data used to make this figure can be found in S1 Data.
(TIF)

S9 Figure **The effects of KDM3A knockdown on the occupancy of Stat1, phosphorylated Stat1, and Brg1 at the GAS of** ***hsp90α*** **.** (**A**) Western blot of the cell extracts from Jurkat cells that were transfected with either the shKDM3A or mock vector using the antibodies shown on the right. GAPDH was used as a control. (**B–F**) ChIP assays. The cells were transfected with KDM3A (i-KDM3A) or GFP shRNA (Mock) and then subjected to ChIP using anti-KDM3A (**B**), anti-Stat1 (**C**), anti-pY-Stat1 (**D**), anti-pS-Stat1 (**D**), or anti-Brg1 (**F**). HS: filled bars; control: open bars. Data are mean ± SD (**p<0.01). The data used to make this figure can be found in S1 Data.
(TIF)

S10 Figure **The effects of MSK1 knockdown on the phosphorylation of KDM3A and the occupancy of Stat1 at the GAS region of** ***hsp90α*** **.** (**A**) The cell extracts from Jurkat cells transfected with either the shMSK1, shGFP or mock vector were used for western blot. Based on western blot for MSK1, only a minimal level of MSK1 was detected in the shMSK1-transfected cells. MSK2 and GAPDH were used as controls. (**B**) The phosphorylation of KDM3A was abolished in H89 (an inhibitor of MSK1)-treated-cells treated with HS (+) or not (−). (**C**) The phosphorylation of KDM3A was induced using anisomycin (+), an activator of MSK1, and was abolished via MSK1 shRNA (i-MSK1)-mediated knockdown. The duration of anisomycin treatment is indicated on top of each lane (min). (**D**) The cells were transfected with MSK1 (i-MSK1) or GFP shRNA (Mock) and then subjected to ChIP using anti-Stat1. HS: filled bars; control: open bars.
(TIF)

S11 Figure **The effects of KDM3A mutants on the occupancy of Stat1 and phosphorylated Stat1 at the GAS region of** ***hsp90α*** **.** (**A**) The Jurkat cells were transfected with western blot of the cell extracts from Jurkat cells that were transfected with either wild type KDM3A, S264A, or S264D mutant of KDM3A using an anti-FLAG antibody. GAPDH was used as a control. (**B–D**) ChIP assays showed the occupancy of Stat1 and phosphorylated Stat1 at the upstream of *hsp90α*.
(TIF)

S12 Figure **The H3K9me2 levels on the promoter of hsp90α, CIITA, and BCL-6 genes.** (**A–D**) The Jurkat (**A and B**) and Raji cells (**C and D**) were treated by heat shock or IFNγ. ChIP assays were performed by using an antibody against H3K9me2, the primers of qPCR were described in Ref [28]. Data are mean ± SD (*p<0.05, **p<0.01). The data used to make this figure can be found in S1 Data.
(TIF)

S13 Figure **Flow chart of the ChIP-seq analysis.**
(TIF)

S1 Table **The ChIP-seq signal peak distributions across the genome.** As controls, two different sets of 7,500 peaks of the same average length and with randomly sampled locations were run, which intersected with the genomic characteristics in the same manner.
(XLSX)

S2 Table **The list of genes with binging peaks (FDR <1×10^−20^) that were subjected to ChIP for KDM3A or p-KDM3A.** Only the peaks in the promoter region (from 4 kb upstream to 2 kb downstream of the TSS) were considered.
(XLSX)

S3 Table **Detailed information for the top statistically valid motifs and the TFs displaying similar motifs based on TOM-TOM.**
(XLS)

S4 Table **The list of p-KDM3A sites displaying the greatest significance in the differences between the HS and control treatments.**
(XLSX)

S5 Table **Primers used in plasmids constructed.**
(DOC)

S6 Table **Primers used in RT-qPCR.**
(DOC)

S7 Table **Primers used in ChIP-qPCR.**
(DOC)

## Abbreviations

AR: androgen receptor
ChIP: chromatin immunoprecipitation
CDK: cyclin-dependent kinases
ChIP-qPCR: ChIP-quantitative PCR
DN: dominant negative
GAS: IFNγ-activated sequence
GO: Gene Ontology
H: histone
GST: Glutathione S-transferase
HS: heat shock
K: lysine
KDM: histone lysine demethylase
MSK1: mitogen- and stress-activated protein kinase 1
p-Ser: pan-phosphorylated serine
RT-qPCR: reverse transcription quantitative PCR
S: serine
TFs: transcription factors
TSS: transcription start site
TTS: transcription terminal site
WCE: whole cell extracts
