# Supplementary figures and images for: Specific Phosphorylation of Histone Demethylase KDM3A Determines Target Gene Expression in Response to Heat Shock
Source: PLoS Biol. 2014 Dec 23;12(12):e1002026. doi: 10.1371/journal.pbio.1002026 (PMC4275180; doi:10.1371/journal.pbio.1002026)

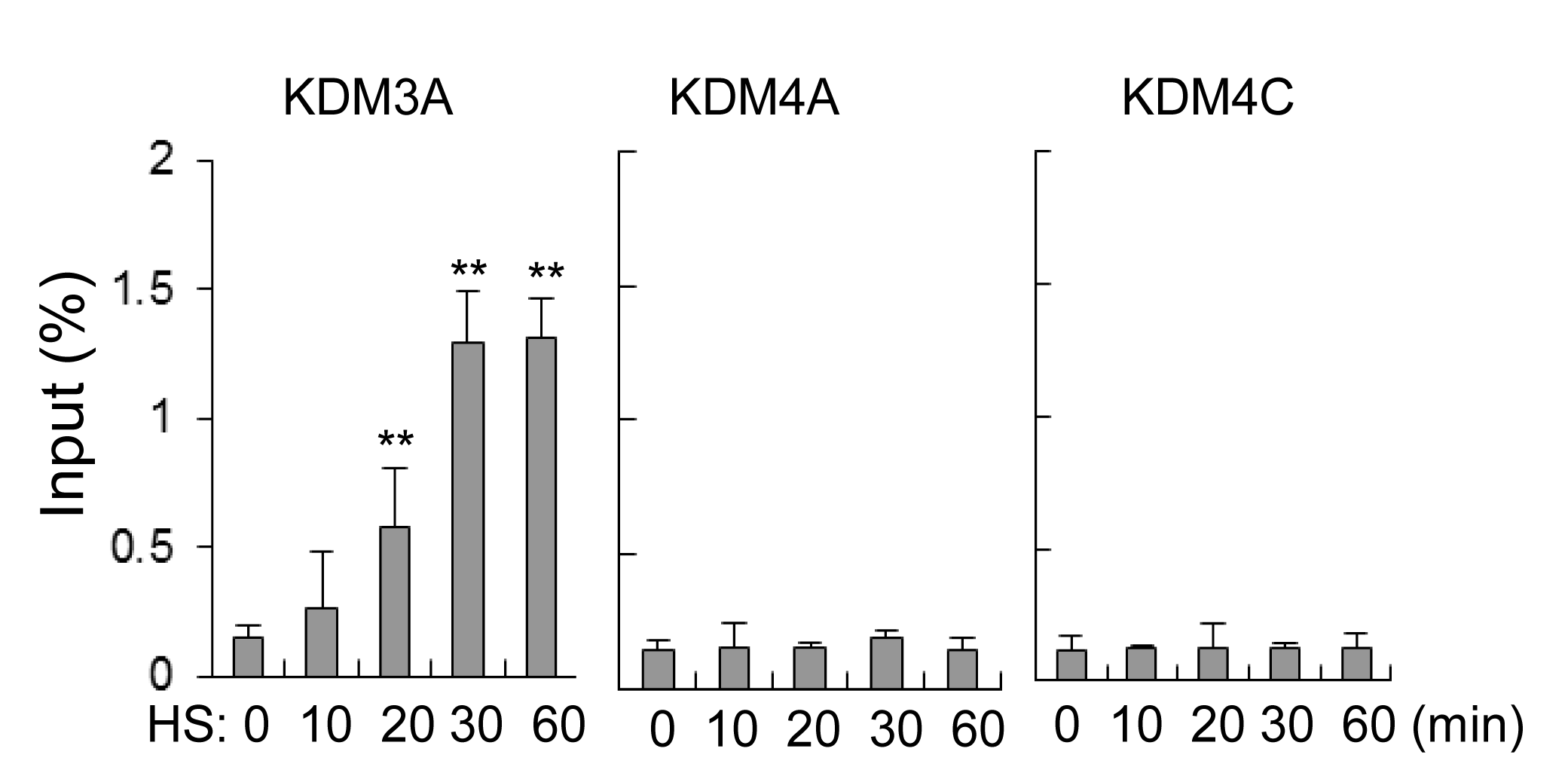

Supplement: S1 Figure — KDM3A is recruited to the upstream of hsp90α in response to heat shock. The ChIP assay demonstrated the recruitment of KDM3A, KDM4A, and KDM4C upstream of human hsp90α upon HS treatment. The cells were transfected with FLAG-tagged KDM3A, KDM4A, or KDM4C. The chromatin fragments were pulled down using a specific antibody against FLAG. The duration of HS treatment is indicated at the bottom of each bar (0–60 min). The annotations are the same as those in Fig. 4B. Data are mean ± SD (*p<0.05, **p<0.01). The data used to make this figure can be found in S1 Data. (TIF) [file pbio.1002026.s002.tif]

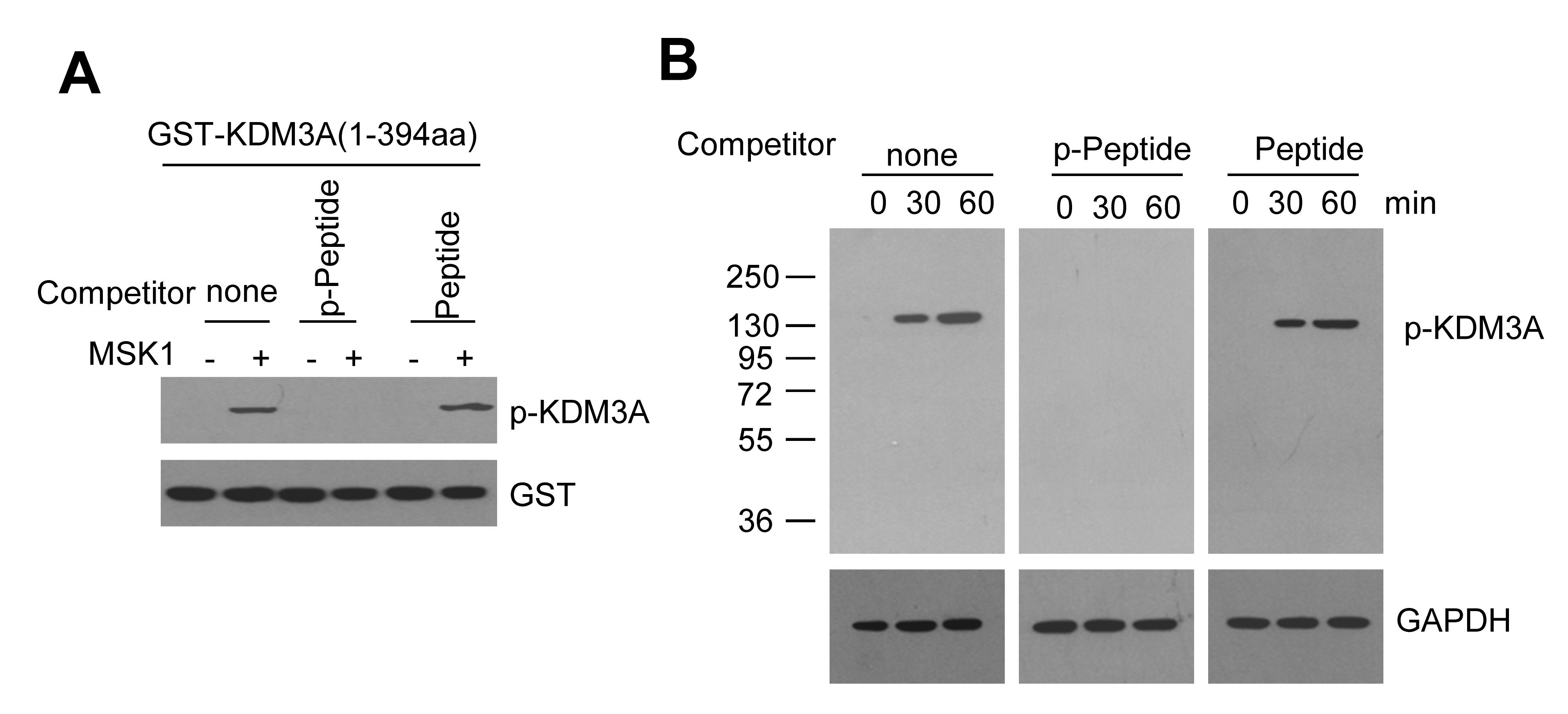

Supplement: S2 Figure — Characterization of the antibody specific for p-KDM3A-S264. (A) Western blot indicating the antibody efficiency for p-KDM3A using KDM3A phosphorylated by MSK1 in vitro. The phosphorylated peptide cVKRK(p)SSENNG (p-peptide) was used as a specific competitor, and the non-phosphorylated peptide was used as a control. (B) The cells were treated with HS for 0, 30, or 60 min. The specificity of the anti-p-KDM3A antibody was determined via western blot, as described above. (TIF) [file pbio.1002026.s003.tif]

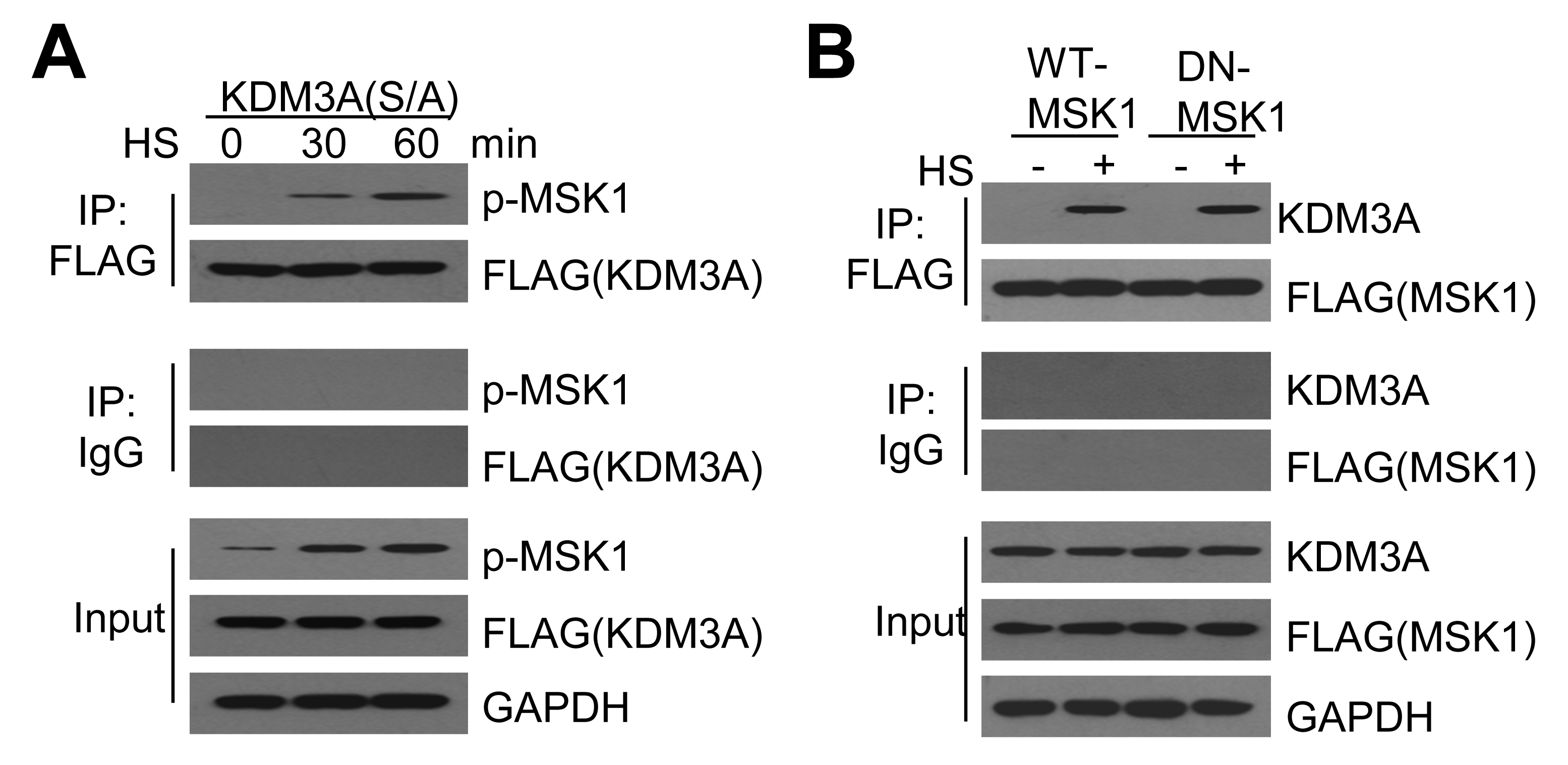

Supplement: S3 Figure — p-KDM3A interacts with MSK1 in heat-shocked cells. (A) The cells were transfected with FLAG-S/A-KDM3A. Co-IP assays were performed using an anti-FLAG antibody, followed by western blot using antibodies for p-MSK1 and FLAG. (B) The cells were transfected with FLAG-tagged wild-type or DN-MSK1. Co-IP was performed using an anti-FLAG antibody, followed by western blot using anti-KDM3A and anti-FLAG antibodies. The inputs and the IP using IgG are shown as controls. (TIF) [file pbio.1002026.s004.tif]

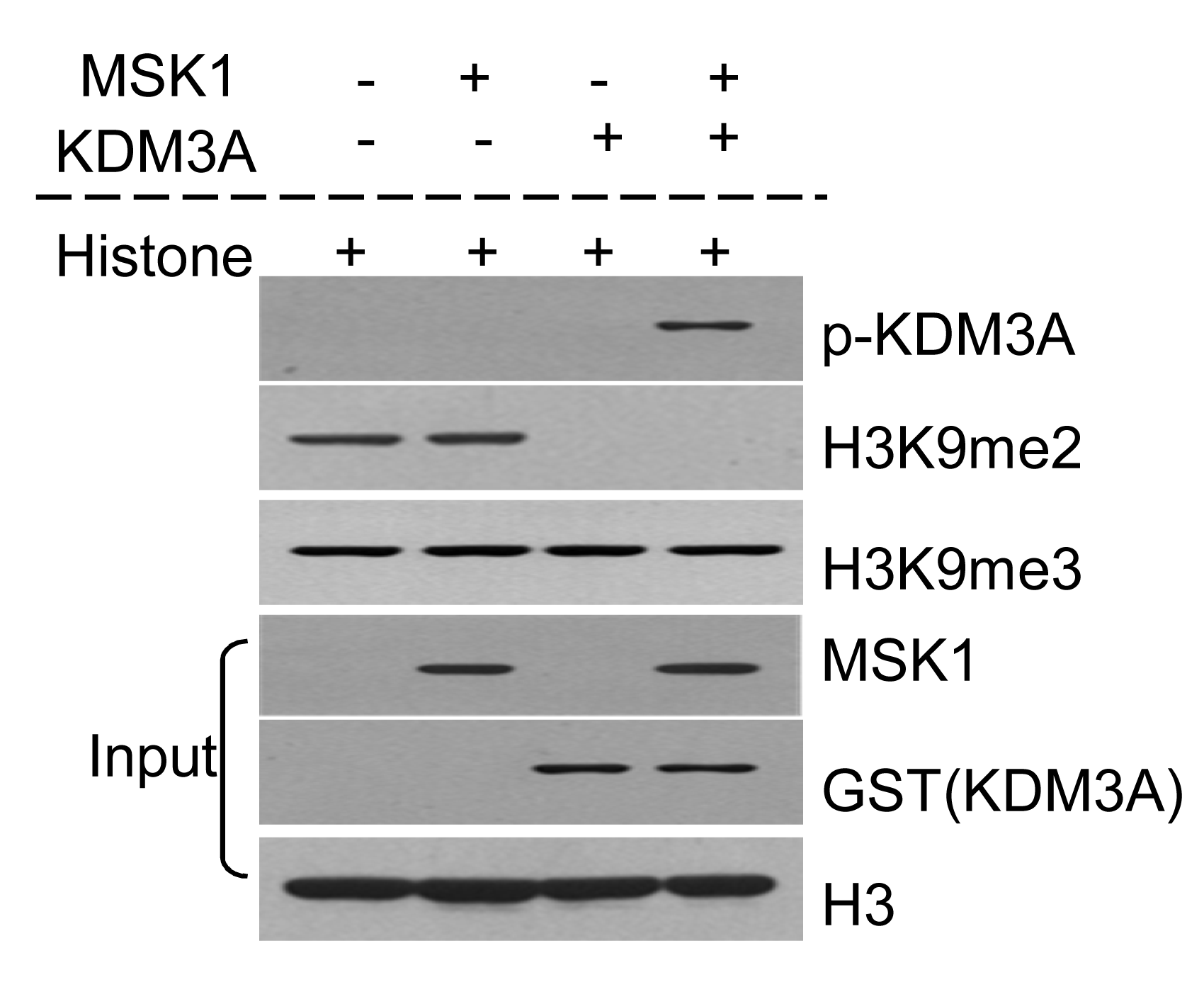

Supplement: S4 Figure — Histone H3K9me2 demethylation assay in vitro. The histone demethylation assay demonstrated that the phosphorylation of KDM3A at S264 did not affect the demethylase activity of KDM3A on H3K9me2. Recombinant MSK1 and GST-KDM3A were initially mixed for the kinase assay and were subsequently added to histones that were purified from HeLa cells for the demethylase activity assay. The reaction products were separated via SDS-PAGE for western blot using the H3K9me2 antibody. Other antibodies used included those used for the kinase assay control: H3K9me3 as a demethylase activity control and MSK1, GST, and H3 as input controls. (TIF) [file pbio.1002026.s005.tif]

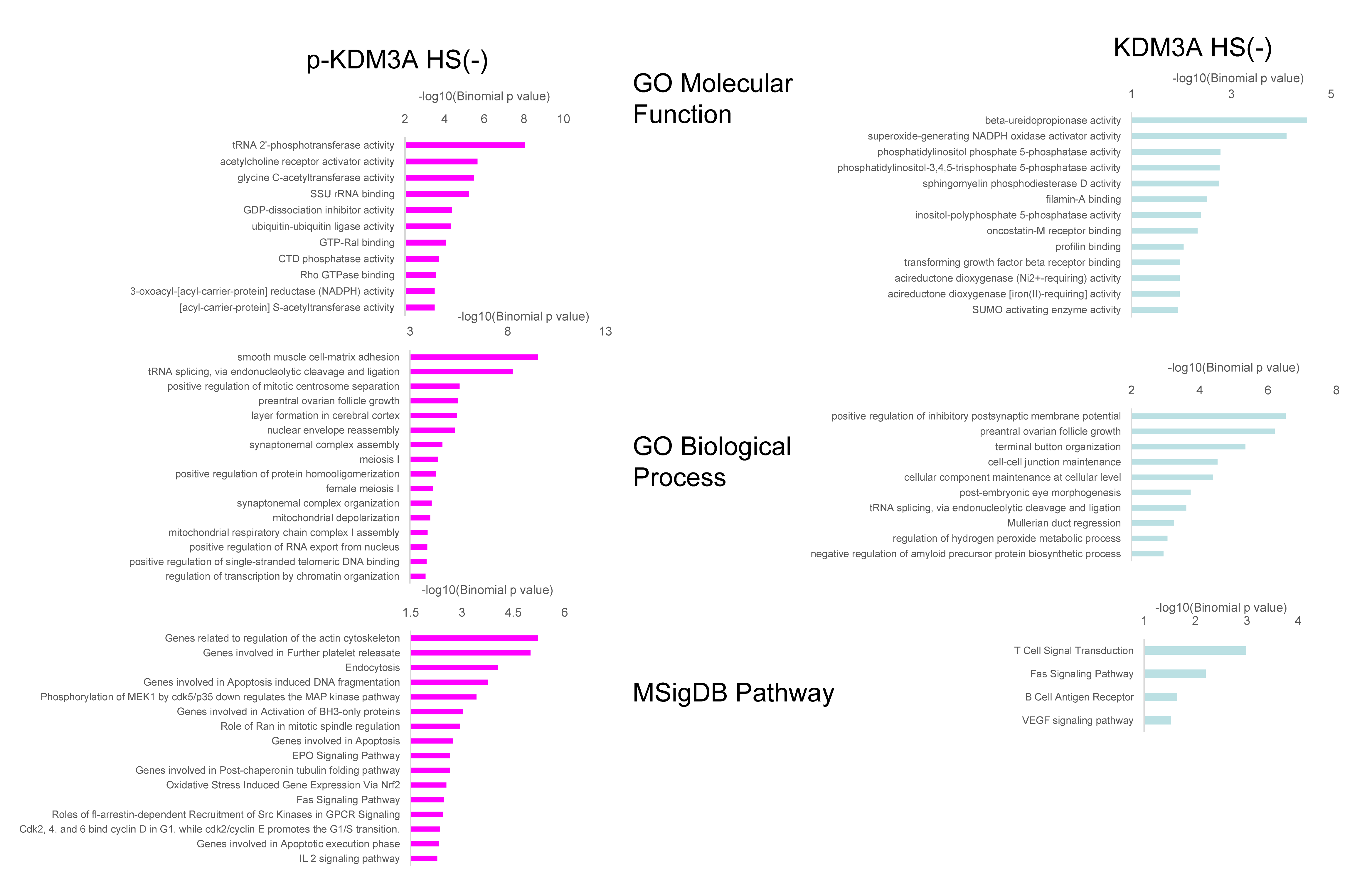

Supplement: S5 Figure — GO and pathway analyses of the KDM3A HS (-) and p-KDM3A HS (-) binding genes. (TIF) [file pbio.1002026.s006.tif]

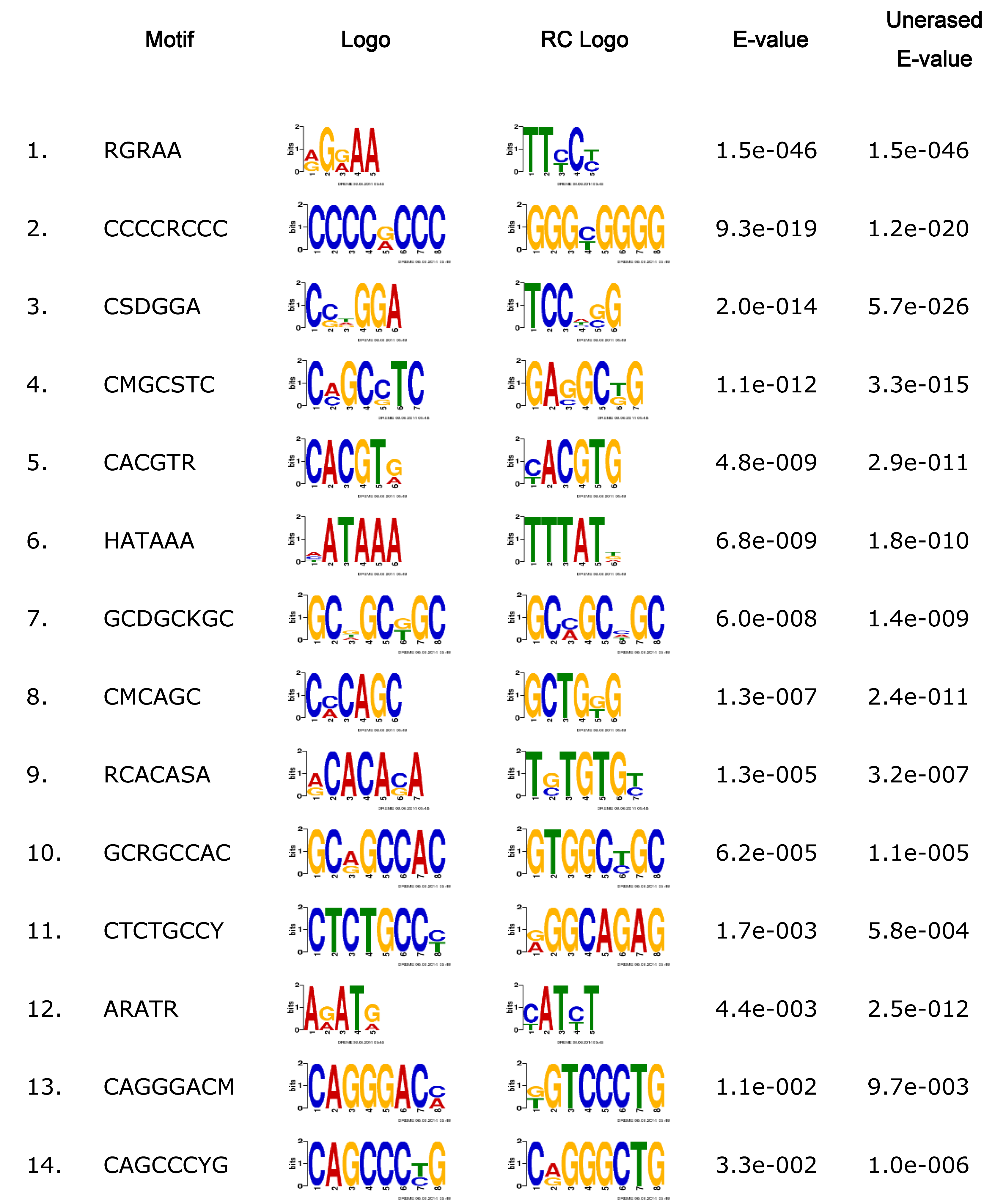

Supplement: S6 Figure — Motif analysis of the p-KDM3A-enriched regions using discriminative DNA motif discovery (DREME) [49]. (TIF) [file pbio.1002026.s007.tif]

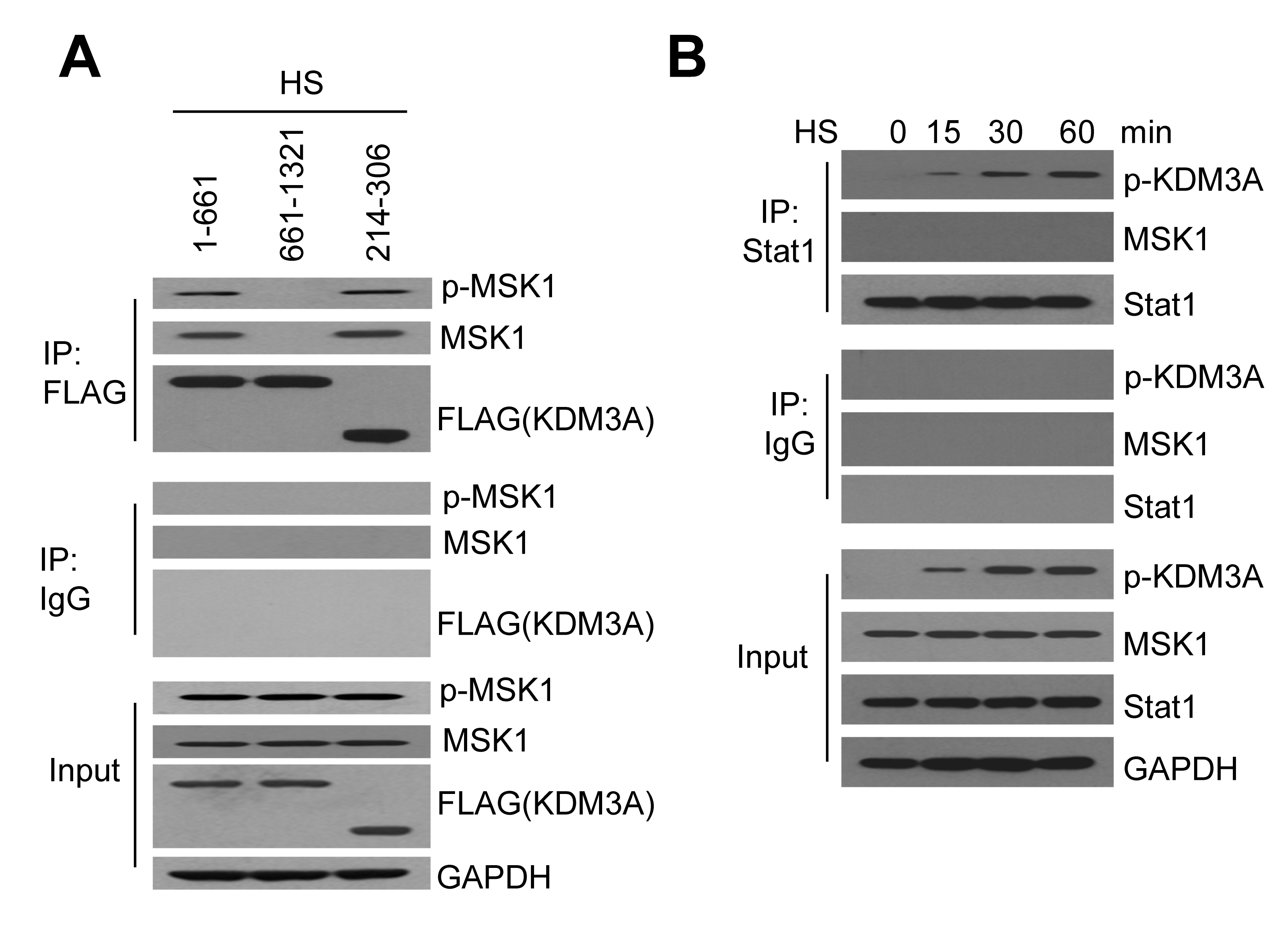

Supplement: S7 Figure — Interaction between Stat1 and p-KDM3A. (A) Jurkat cells were transfected with FLAG-KDM3A(1-661), FLAG-KDM3A(661-1321) and FLAG-KDM3A(214-306) and treated with HS for 1 hr. Co-IP assays were performed using an anti-FLAG antibody, followed by western blot using antibodies for p-MSK1, MSK1, and FLAG. (B) The cells were treated with HS for the indicated time (min). Then, the cell lysates were immunoprecipitated using an anti-Stat1 antibody, followed by western blot using antibodies against Stat1, MSK1, and p-KDM3A. The inputs and IP using IgG are shown as controls. (TIF) [file pbio.1002026.s008.tif]

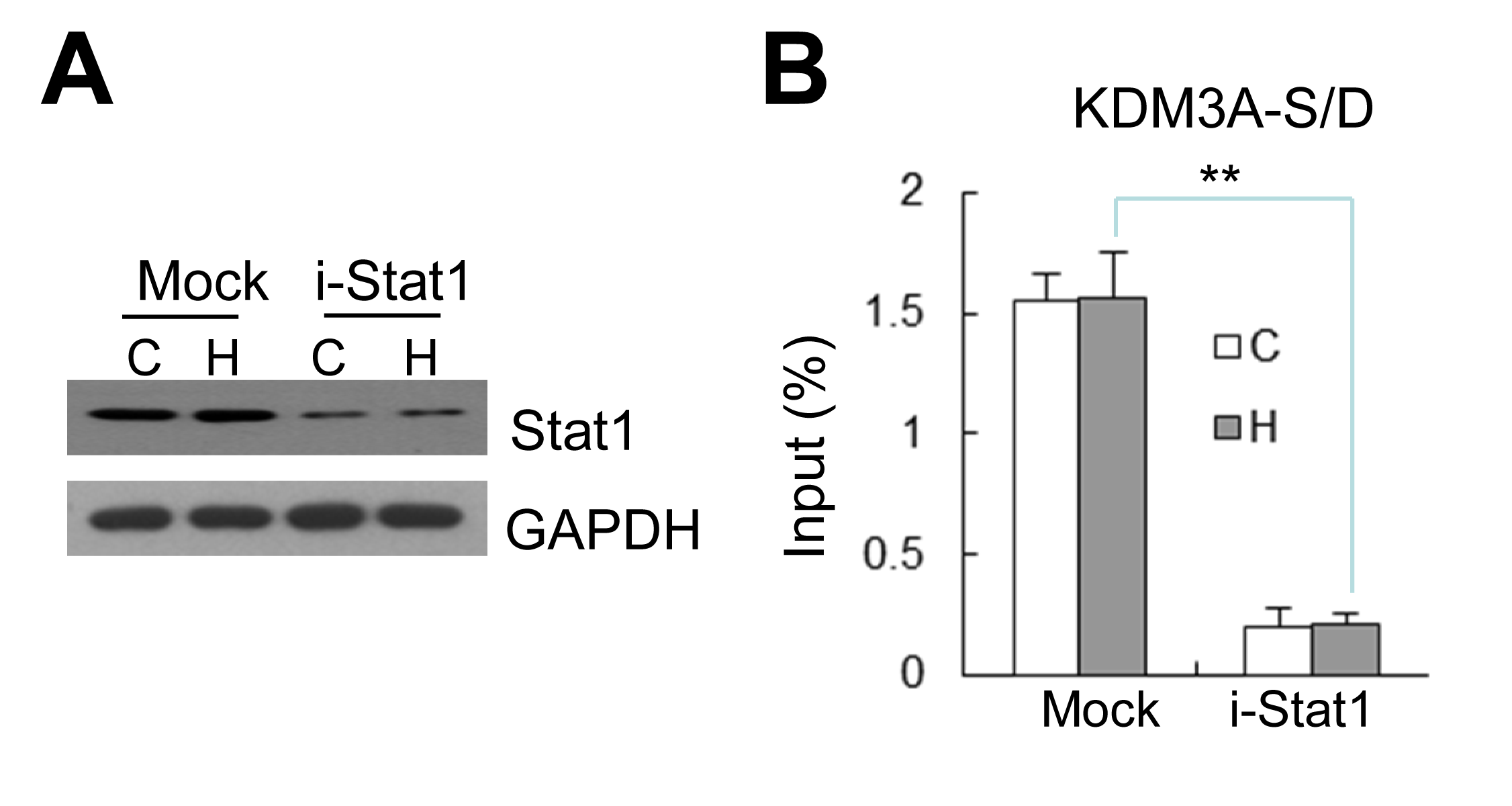

Supplement: S8 Figure — The effects of Stat1 knockdown on the occupancy of phosphorylation mimic of KDM3A. (A) The cell extracts from Jurkat cells transfected with either the i-Stat1 or mock vector were used for western blot. Based on western blot for Stat1, only a minimal level of Stat1 was detected in the i-Stat1-transfected cells. GAPDH was used as a control. (B) The Jurkat cells were co-transfected with KDM3A-S/D and Mock or i-Stat1. A ChIP assay showed the effect of knockdown of Stat1 on the occupancy of KDM3A-S/D at the upstream of hsp90α. Data are mean ± SD (**p<0.01). The data used to make this figure can be found in S1 Data. (TIF) [file pbio.1002026.s009.tif]

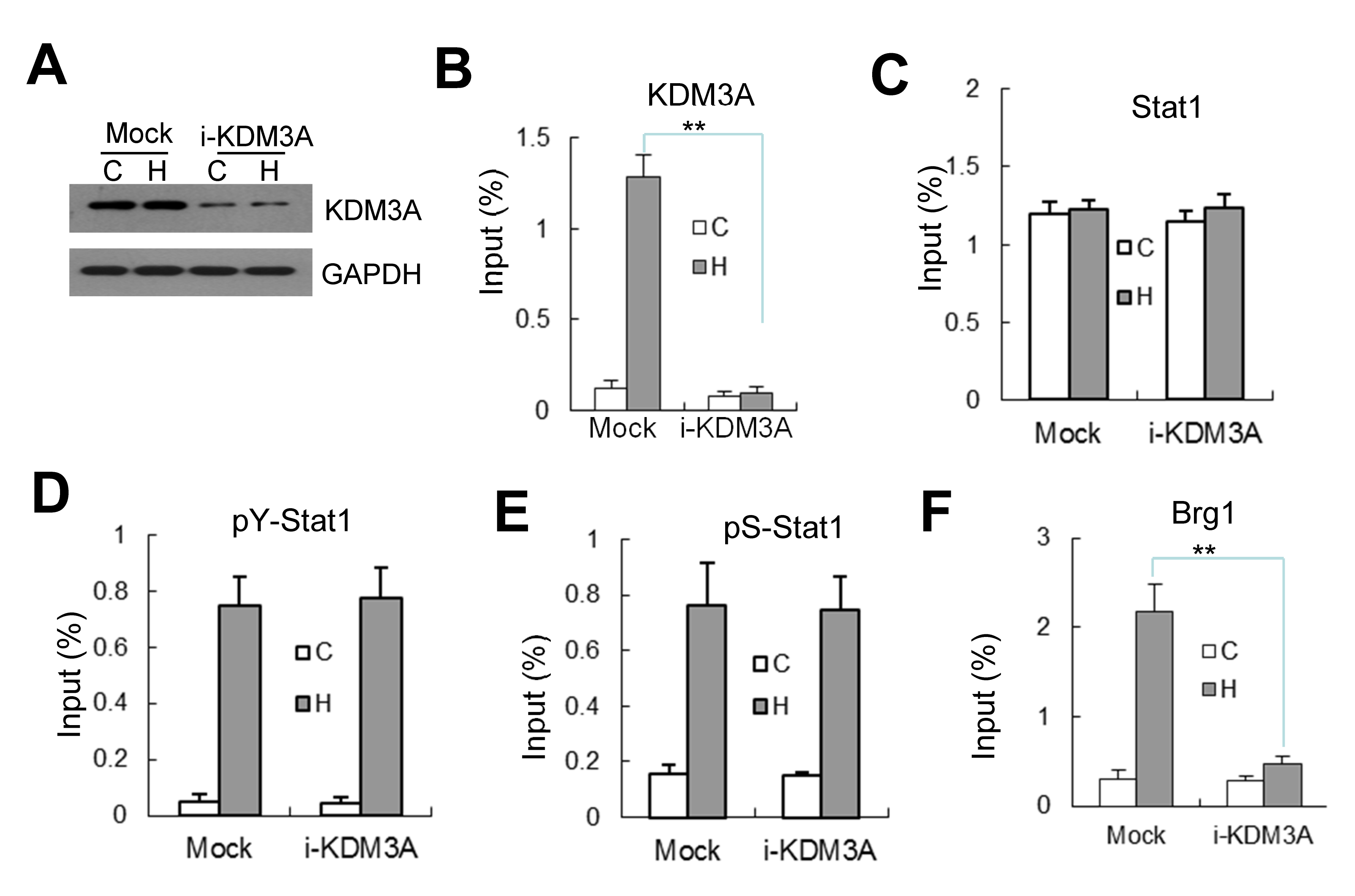

Supplement: S9 Figure — The effects of KDM3A knockdown on the occupancy of Stat1, phosphorylated Stat1, and Brg1 at the GAS of hsp90α . (A) Western blot of the cell extracts from Jurkat cells that were transfected with either the shKDM3A or mock vector using the antibodies shown on the right. GAPDH was used as a control. (B–F) ChIP assays. The cells were transfected with KDM3A (i-KDM3A) or GFP shRNA (Mock) and then subjected to ChIP using anti-KDM3A (B), anti-Stat1 (C), anti-pY-Stat1 (D), anti-pS-Stat1 (D), or anti-Brg1 (F). HS: filled bars; control: open bars. Data are mean ± SD (**p<0.01). The data used to make this figure can be found in S1 Data. (TIF) [file pbio.1002026.s010.tif]

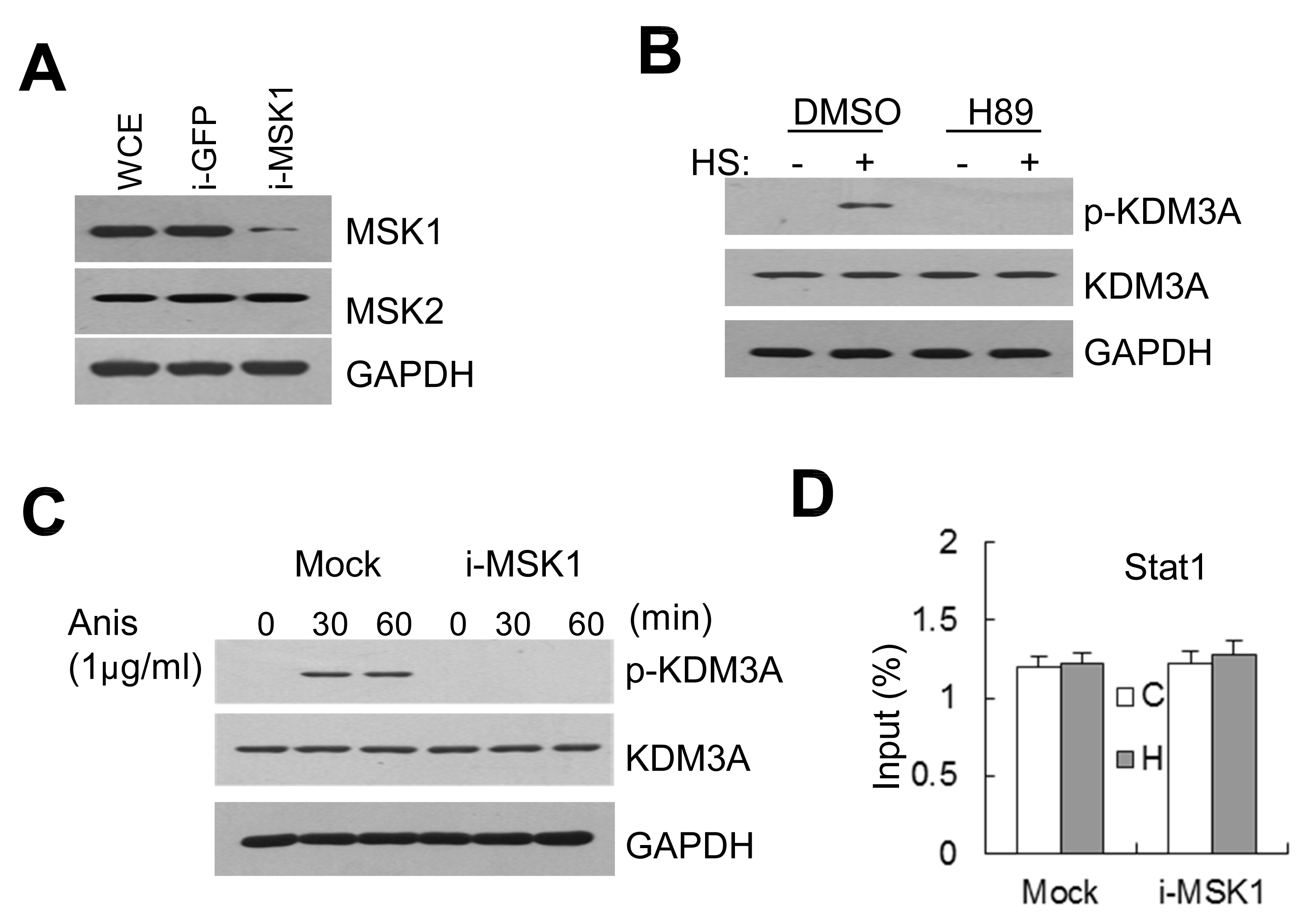

Supplement: S10 Figure — The effects of MSK1 knockdown on the phosphorylation of KDM3A and the occupancy of Stat1 at the GAS region of hsp90α . (A) The cell extracts from Jurkat cells transfected with either the shMSK1, shGFP or mock vector were used for western blot. Based on western blot for MSK1, only a minimal level of MSK1 was detected in the shMSK1-transfected cells. MSK2 and GAPDH were used as controls. (B) The phosphorylation of KDM3A was abolished in H89 (an inhibitor of MSK1)-treated-cells treated with HS (+) or not (−). (C) The phosphorylation of KDM3A was induced using anisomycin (+), an activator of MSK1, and was abolished via MSK1 shRNA (i-MSK1)-mediated knockdown. The duration of anisomycin treatment is indicated on top of each lane (min). (D) The cells were transfected with MSK1 (i-MSK1) or GFP shRNA (Mock) and then subjected to ChIP using anti-Stat1. HS: filled bars; control: open bars. (TIF) [file pbio.1002026.s011.tif]

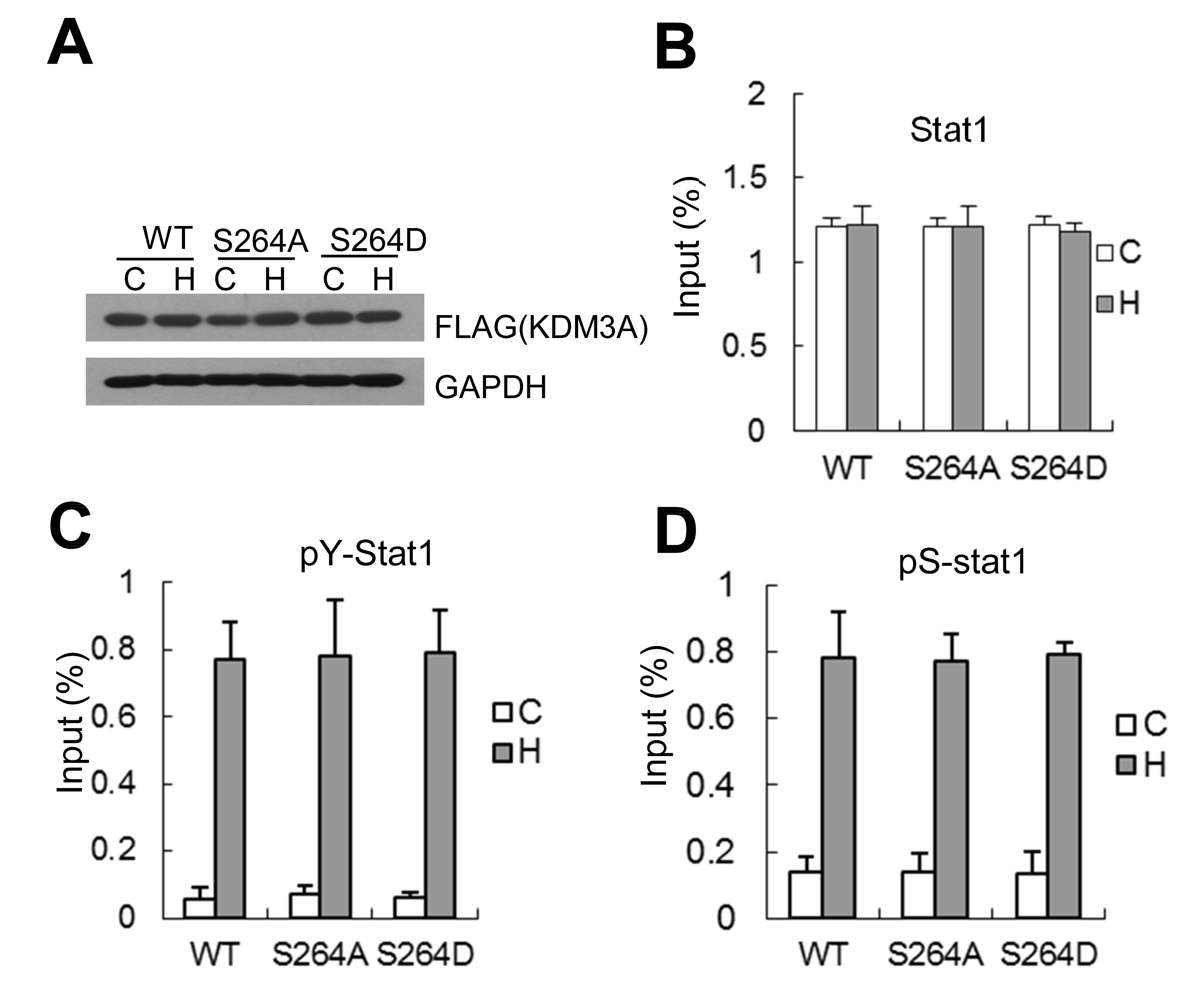

Supplement: S11 Figure — The effects of KDM3A mutants on the occupancy of Stat1 and phosphorylated Stat1 at the GAS region of hsp90α . (A) The Jurkat cells were transfected with western blot of the cell extracts from Jurkat cells that were transfected with either wild type KDM3A, S264A, or S264D mutant of KDM3A using an anti-FLAG antibody. GAPDH was used as a control. (B–D) ChIP assays showed the occupancy of Stat1 and phosphorylated Stat1 at the upstream of hsp90α. (TIF) [file pbio.1002026.s012.tif]

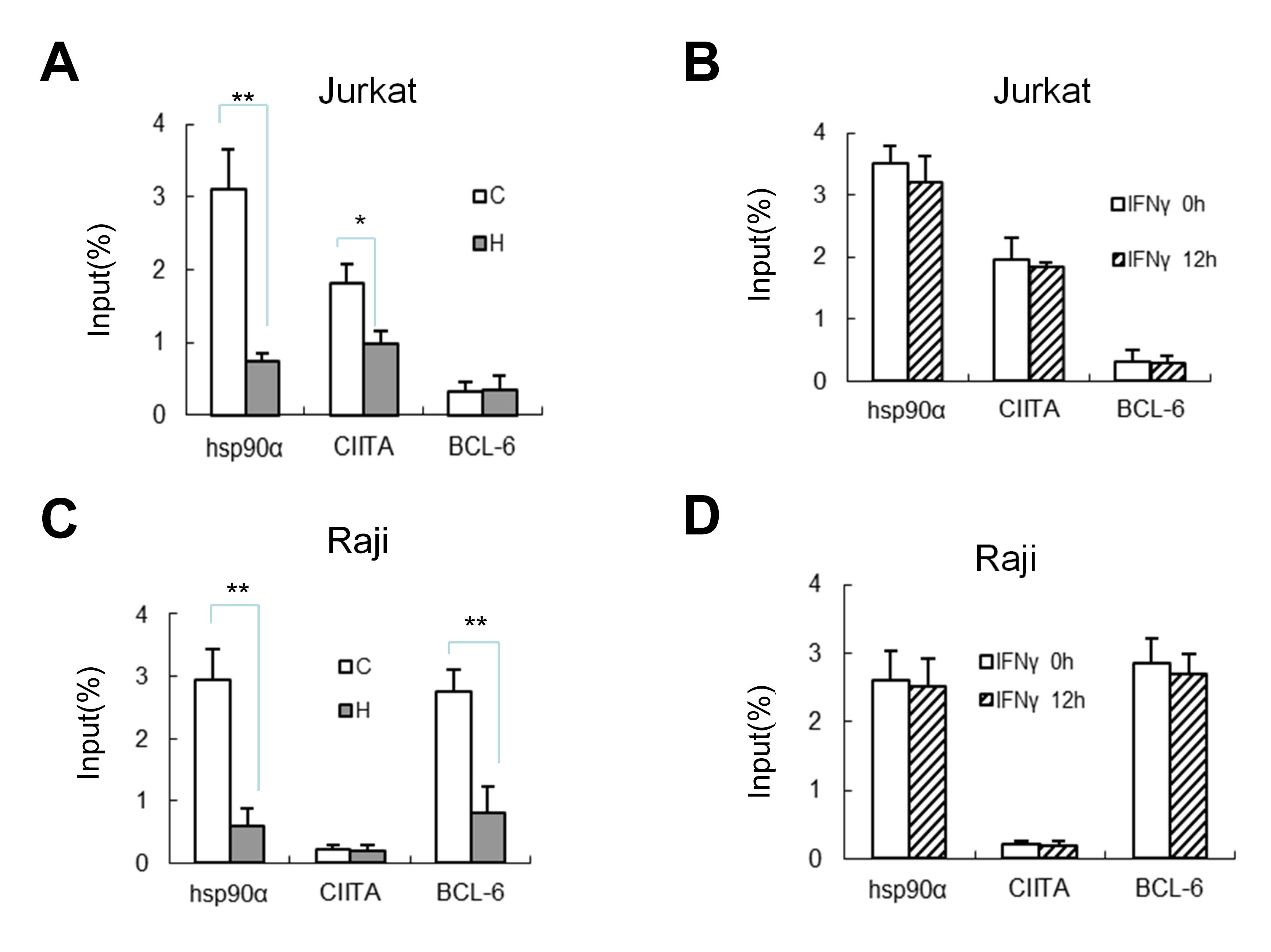

Supplement: S12 Figure — The H3K9me2 levels on the promoter of hsp90α, CIITA, and BCL-6 genes. (A–D) The Jurkat (A and B) and Raji cells (C and D) were treated by heat shock or IFNγ. ChIP assays were performed by using an antibody against H3K9me2, the primers of qPCR were described in Ref [28]. Data are mean ± SD (*p<0.05, **p<0.01). The data used to make this figure can be found in S1 Data. (TIF) [file pbio.1002026.s013.tif]

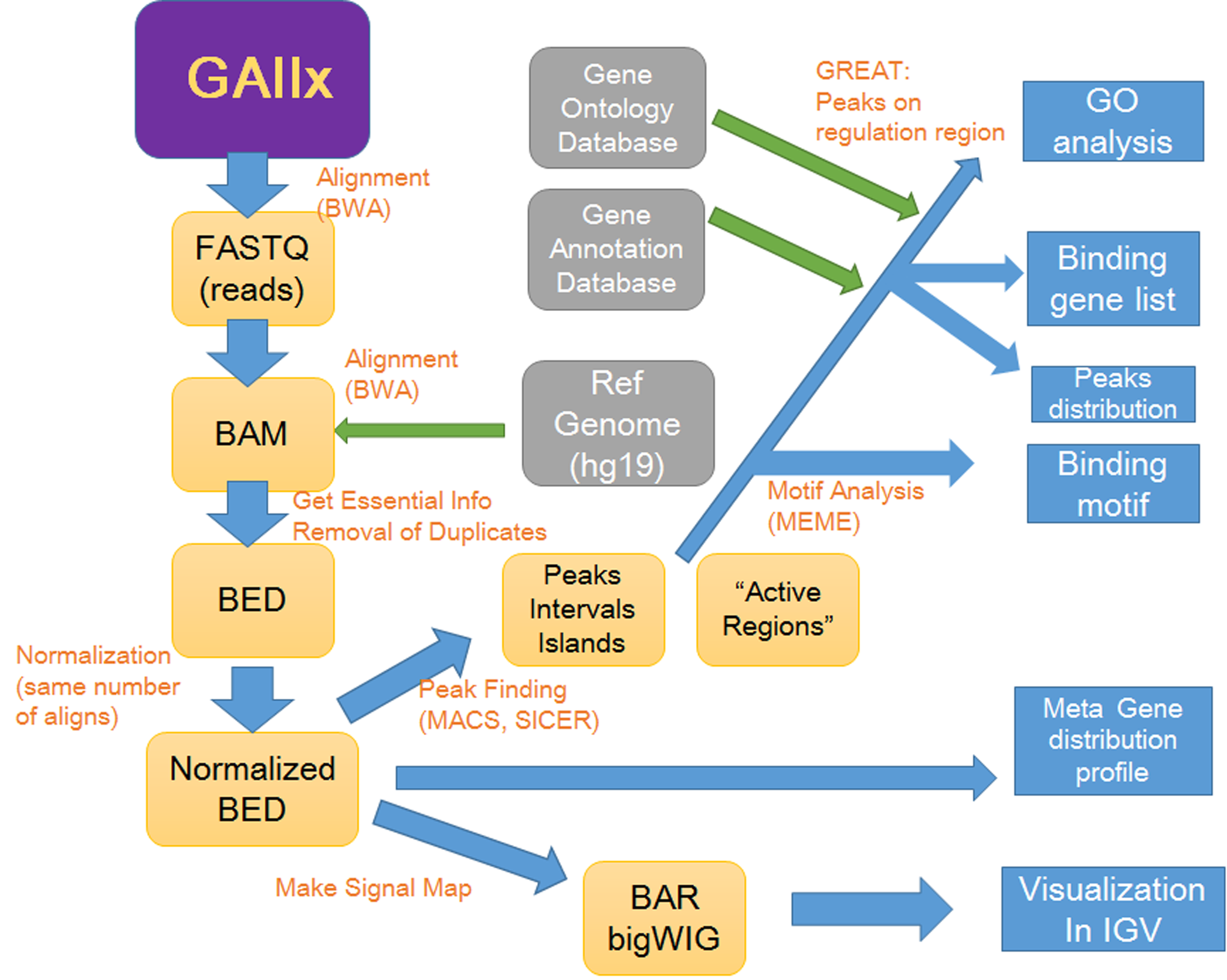

Supplement: S13 Figure — Flow chart of the ChIP-seq analysis. (TIF) [file pbio.1002026.s014.tif]
