# Supplementary material for: Specific Phosphorylation of Histone Demethylase KDM3A Determines Target Gene Expression in Response to Heat Shock
Source: PLoS Biol. 2014 Dec 23;12(12):e1002026. doi: 10.1371/journal.pbio.1002026 (PMC4275180; doi:10.1371/journal.pbio.1002026)
Supplement: S5 Table — Primers used in plasmids constructed. (DOC) [file pbio.1002026.s019.doc]

**Table S5. Primers used in plasmids constructed.**

|  | forward oligonucleotide（5′-3′） | reverse oligonucleotide（5′-3′） |
| --- | --- | --- |
| WT-MSK1 | CGGAATTCGCCGGAAAAAAAGGAGAAGATGG | GCTCTAGAGATTCCAATGAGACCAACGG |
| MSK1-D195A | CATGTGGTGCTGACAGCTTTTGGTCTGAGTAAG | CTTACTCAGACCAAAAGCTGTCAGCACCACATG |
| MSK1-D565A | GAAATTAAAATAATTGCTTTTGGATTTGCACGG | CCGTGCAAATCCAAAAGCAATTATTTTAATTTC |
| KDM3A-S264A | GCTGTAAAACGCAAGGCTTCTGAGAATAATG | CATTATTCTCAGAAGCCTTGCGTTTTACAGC |
| KDM3A-S265A | GTAAAACGCAAGTCTGCTGAGAATAATGGAA | TTCCATTATTCTCAGCAGACTTGCGTTTTAC |
| KDM3A-S445A | CTAGAACATGCACCTGCCCCATCGGATGTTT | AAACATCCGATGGGGCAGGTGCATGTTCTAG |
| KDM3A-S463A | GGTGTCAATAGTGATGCCCCTAATAACTGTTC | GAACAGTTATTAGGGGCATCACTATTGACACC |
| KDM3A(214-306） | CGCGGATCCGGAAACCCAGCATCAAA | CCGGAATTCGCCGCAGTACAGCCAA- |
| Stat1（317-750） | CGCGGATCCACAGGGGTCCAGTTCACTGT | CCGGAATTCCTATACTGTGTTCATCATACTG |
| Stat1（129-235） | CGCGGATCCATATTCAGAGCACAGTGA | CCGGAATTCTTCATCATTAATCAGGGC |
| Stat1（231-317） | CGCGGATCCAGCTGATTAATGATGAACTAG | CCGGAATTCGAAACGAGCTCTGAATGA |
