# Supplementary material for: Specific Phosphorylation of Histone Demethylase KDM3A Determines Target Gene Expression in Response to Heat Shock
Source: PLoS Biol. 2014 Dec 23;12(12):e1002026. doi: 10.1371/journal.pbio.1002026 (PMC4275180; doi:10.1371/journal.pbio.1002026)
Supplement: S6 Table — Primers used in RT-qPCR. (DOC) [file pbio.1002026.s020.doc]

**Table S6. Primers used in qRT-PCR.**

|  | forward oligonucleotide (5`-3`) | reverse oligonucleotide (5`-3`) |
| --- | --- | --- |
| DNAJB1 | CTACCACCCGGACAAGAACAA | CCACTCCCCTTTAGGCCTTC |
| SERPINH1 | GGCAGTGGAGAACATCCTGGTG | GCCGTGGAGTTGCTGAGTGA |
| SMIM20 | GCCGCCTTCTATCCCATCT | CCTTCTTGTACTCCTCCAATCTCA |
| RNASEK | ATGGCGTCGCTCCTGTGCT | GCTGACTTGCTCGTAAAGGTTGTAT |
| HSP90AA1 | CTGGCAATGGCAGAAACTG | GAATCCGGAAGCAGGAAGAG |
| GAPDH | GAAGGTGAAGGTCGGAGTC | GAAGATGGTGATGGGATTT |
