# Supplementary material for: Specific Phosphorylation of Histone Demethylase KDM3A Determines Target Gene Expression in Response to Heat Shock
Source: PLoS Biol. 2014 Dec 23;12(12):e1002026. doi: 10.1371/journal.pbio.1002026 (PMC4275180; doi:10.1371/journal.pbio.1002026)
Supplement: S7 Table — Primers used in ChIP-qPCR. (DOC) [file pbio.1002026.s021.doc]

**Table S7. Primers used in ChIP-qPCR.**

|  | forward oligonucleotide (5`-3`) | reverse oligonucleotide (5`-3`) | Location to the TSS |
| --- | --- | --- | --- |
| DNAJB1 | GCCAGCGTGCCTCAGTTTCC | TACGACGTGCTCAGCGACCC | 193~317 |
| SERPINH1 | TAGCATCATGCACATATCGAGGAA | GGCGAAGCAACTACCCAACC | 2156~2461 |
| SMIM20 | TTTCGGCGGCTTCATCTCC | GTTCTTTCCACGGCACCAC | 179~319 |
| RNASEK | TCTTCCAATACTCACGCCCTAG | CAGGAGCGACGCCATAAAG | -42~220 |
| HSP90AA1 | CTGGCAATGGCAGAAACTG | GAATCCGGAAGCAGGAAGAG | -1667~-1507 |
